# A key regulator of missing-self innate immunity is polymorphic and under diversifying selection

**DOI:** 10.1093/molbev/msag082

**Published:** 2026-04-06

**Authors:** Rocco F Notarnicola, Magdalena Herdegen-Radwan, Joanna Różańska-Wróbel, Mateusz Konczal, Karolina Przesmycka, Petr Kotlík, Wiesław Babik, Jacek Radwan

**Affiliations:** Evolutionary Biology Group, Adam Mickiewicz University, Poznań, Poland; Department of Behavioural Ecology, Adam Mickiewicz University, Poznań, Poland; Evolutionary Biology Group, Adam Mickiewicz University, Poznań, Poland; Evolutionary Biology Group, Adam Mickiewicz University, Poznań, Poland; Evolutionary Biology Group, Adam Mickiewicz University, Poznań, Poland; Centre for Ecology and Conservation, University of Exeter, Penryn, UK; Laboratory of Molecular Ecology, Institute of Animal Physiology and Genetics of the Czech Academy of Sciences, Liběchov, Czech Republic; Faculty of Biology, Jagiellonian University, Institute of Environmental Sciences, Kraków, Poland; Evolutionary Biology Group, Adam Mickiewicz University, Poznań, Poland

**Keywords:** missing-self, innate immunity, polymorphism, complement factor H, host-parasite co-evolution, genomic scan of selection

## Abstract

Host–parasite co-evolution drives the diversification of host immune genes involved in the recognition of pathogen antigens and molecular patterns. In contrast, the immune genes involved in self-recognition and inhibition of immune responses against self-cells (missing-self immunity) may be evolutionarily constrained by the need to interact with self-components. However, many pathogens, such as the Lyme disease agent *Borrelia*, hijack these genes to evade the immune system and may therefore select for their diversification. How these contrasting but concurrent selective forces shape the evolution of missing-self regulators is not clearly understood. To fill this gap, we investigated polymorphism and molecular signatures of selection acting on a missing-self regulator, the complement factor H (CFH), in bank vole populations, which are an important wild reservoir for *Borrelia*. We then compared the geographic structuring in the CFH domain interacting with *Borrelia* (CCP 20) against a genomic background represented by RAD-seq markers. We found signals of positive and diversifying selection at CCP 20, suggesting that CFH evolved in response to pressures from pathogens. Additionally, we found other innate immunity genes within the alternative complement pathway, which is regulated by CFH, under diversifying selection, highlighting its involvement in host–parasite coevolution. This study demonstrates that an innate missing-self sensor in a wild vertebrate is under diversifying selection, likely driven by pathogens.

## Introduction

Immune genes are often found to evolve at faster rates than the genomic average, likely in response to ongoing adaptation of ever-evolving pathogens (Obbard et al. 2009; Enard et al. 2016; Shultz and Sackton 2019). Host immune genes coding for effectors interacting with parasites, such as the major histocompatibility complex (MHC) of the adaptive immunity, or the pattern recognition receptors of the innate immunity, are important components of these dynamics. Indeed, MHC genes encoding proteins crucial in self/non-self-recognition, initiating the adaptive immune response, have become a paradigm for host-pathogen coevolution. They are characterized by high amino acid substitution rates at regions involved in binding to antigens (Hughes and Nei 1988, 1989; Garrigan and Hedrick 2003) and extreme polymorphism, likely maintained by balancing selection imposed by parasites (Apanius et al. 1997; Radwan et al. 2020; Migalska et al. 2022). Selection pressures on innate immunity genes have been relatively less studied, but the evidence is accumulating for fast molecular evolution in the regions involved in interactions with pathogens (Wlasiuk et al. 2009; Tschirren et al. 2013; Tian et al. 2018; Lundberg et al. 2020; Nandakumar et al. 2023). These studies mainly investigated genes encoding proteins that recognize pathogen-associated molecular patterns (PAMPs), while research exploring the evolution of innate immunity genes that mediate the missing-self signal is scant.

Missing identity controls the activity of the alternative complement pathway, an ancient system shared by vertebrates with ascidians and cephalochordates (Nonaka and Kimura 2006). The complement system consists of multiple proteins involved in the phagocytosis and clearance of pathogens and damaged cells, the induction of inflammation, and the regulation of innate and adaptive immunity (Reis et al. 2019). Unlike the classical and lectin pathways, the alternative pathway is in a constant low-level activation state, and destruction of healthy host cells is prevented by protein regulators (Reis et al. 2019). One such regulator is the complement factor H (CFH), a 155 kDa protein with conserved structure and function (Schmidt et al. 2008; Pouw et al. 2015). The CFH gene consists of 22 exons that form 20 complement control protein (CCP) domains, also known as Short Consensus Repeats or *sushi*. The 20 domains contain several glycosylation sites and binding sites to polyanions and to complement Component 3b (C3b; Schmidt et al. 2008; Pouw et al. 2015). The binding of CFH leads to C3b inactivation and/or prevents the formation of the C3 convertase (Meri et al. 2013; Schmidt et al. 2016), a protein that initiates the complement cascade that results in the opsonization and destruction of pathogens. Host cells are protected from complement activation via binding of CFH to polyanions (sialic acids, glycosaminoglycans, phospholipids), molecules present on vertebrate cells but not on microorganisms (Meri 2016)—therefore, bound CFH represents a self-identity signal. However, some pathogens, including *Borrelia burgdorferi*, *Pseudomonas aeruginosa*, and *Haemophilus influenzae,* evolved surface proteins that bind to CFH and thus hijack this self-identity signal (Meri et al. 2013). Although CFH evolution might be constrained by the need to effectively bind to self-motifs, hijacking by pathogens may trigger its adaptive evolution (Carrillo-Bustamante et al. 2016). However, how CFH evolves in response to pathogens is not well understood.

Many of the bacterial proteins that hijack CFH to mimic self and evade complement activation, including *Borrelia*'s outer surface protein E (OspE), bind to CCP domain 20 (Bhattacharjee et al. 2013; Meri et al. 2013), which also contains polyanion binding sites (Meri 2016) and may therefore be subject to complex evolutionary processes/pressures. Cagliani et al. (2016) analyzed molecular evolution of the complement system among primates and identified signals of positive selection in regions, such as CCP 6-7 and 19-20 of CFH, involved in interactions with pathogens belonging to different phyla. Furthermore, using *in-silico* structural modeling, the authors showed that *Borrelia*'s OspE residues interacting with CCP 20 were themselves under positive selection. However, the relevance of these interactions to the evolution of CFH is unclear, given that there is no evidence that *Borrelia* can naturally infect primates (Wolcott et al. 2021), while humans are only incidental and dead-end hosts, from which transmission to other competent hosts is interrupted. In captive rhesus macaques, intraspecific variation within CCP 6 was linked to the binding efficiency of a meningococcal protein that evades immune recognition (Konar et al. 2015). Additionally, in humans, mutations in CCP 19-20 were linked to several autoimmune diseases, such as age-related macular degeneration (Rodriguez et al. 2014; Ferluga et al. 2017), highlighting possible constraints on CFH evolution imposed by its involvement in self-recognition. Whether such constraints restrict polymorphism at CFH is unknown, as, to our knowledge, no study systematically investigated CFH polymorphism in any wild populations. Overall, it remains unclear how the contrasting selective pressures, exerted by pathogens and the role in self-recognition, shape CFH evolution.

Here, we characterize CFH polymorphism and analyze signatures of selection in wild populations of bank voles (*Clethrionomys glareolus*), a small woodland rodent with widespread distribution in Europe (Kotlik et al. 2006). After the last glacial maximum (LGM), *C. glareolus* colonized Europe from several refugia, which included Carpathian and Eastern refugia, giving rise to contemporary populations that came back into contact in central Poland (Wójcik et al. 2010; Marková et al. 2020). A third lineage (Western) was also identified in previous studies, though it contributes some ancestry primarily in southern Poland (Wójcik et al. 2010; Marková et al. 2020). The analysis of genetic differentiation based on heart transcriptomes between 2 populations representing these 2 post-glacial colonization areas revealed CFH as one of the most divergent genes between the 2 lineages (*d*_xy_ = 0.017; max *F*_STnonsyn_ = 1; mean *F*_ST_ = 0.72; Niedziałkowska et al. 2023; Figure S1). This polymorphism may be functionally relevant given that the bank vole is one of the main reservoirs for *Borrelia burgdorferi* sensu *lato* in Europe (Humair et al. 1999; van Duijvendijk et al. 2015). However, the analyses in Niedziałkowska et al. (2023) were based only on 18 individuals from 2 populations (one population per post-glacial colonization area), therefore their results may represent an effect of sampling rather than regional structuring. Furthermore, no formal tests for signals of selection acting on the polymorphic sites in CFH were conducted.

In this study, we analyze CFH polymorphism within and across 13 bank vole populations spanning the 2 major post-glacial colonization areas (Eastern and Carpathian). We implement 2 approaches to understand the nature of selection shaping polymorphism in this gene. The first one relies on the relative rate of non-synonymous to synonymous substitutions, which can detect positive selection over the history of the species (Garrigan and Hedrick 2003). The second approach investigates how contemporary selection shapes the differentiation of allele frequencies between populations. Loci under balancing selection tend to remain polymorphic within populations, resulting in lower differentiation of allele frequencies between populations (Schierup et al. 2000). In contrast, local adaptation leads to stronger population structure and higher allele frequency differentiation in loci under selection compared to the genomic average. For this second approach, we conducted robust outlier tests with BayPass, which factors in complex demographic histories (shared ancestry and gene flow) between populations (Günther and Coop 2013; Gautier 2015) and compared the extent/levels of CFH differentiation across populations with genome-wide differentiation assessed by RAD-seq markers. Additionally, we checked whether the genes in proximity to outlier RAD markers were involved with immunity, particularly with the complement pathway.

## Results

### CFH is polymorphic and under positive selection, particularly at the CCP 20 domain

**Figure 1:**
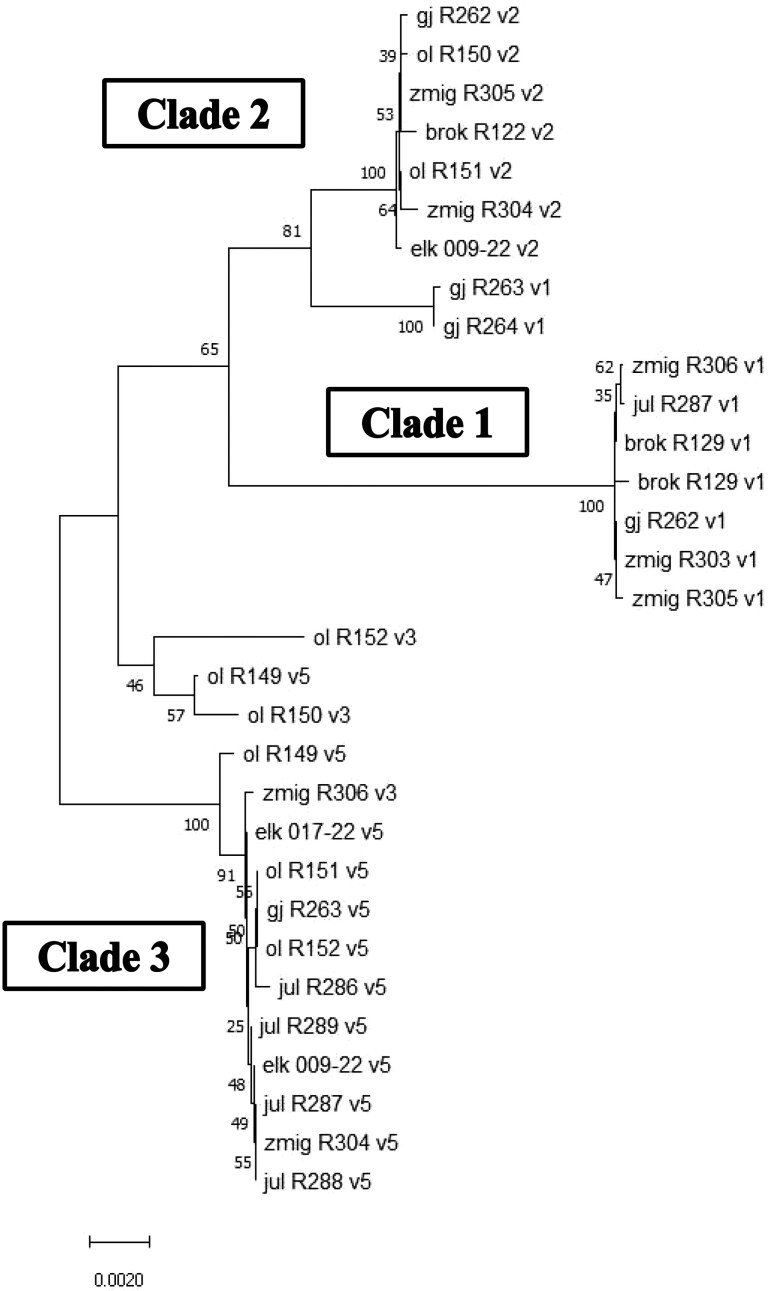
The full-length CFH sequences group into 3 main clades. Neighbor-joining tree of the full-length CFH sequences generated with MEGA11 using the Tamura 3-parameter model. Sample IDs show population of origin, individual ID, and homology of the last exon with the CCP 20 variants (v1, v2, v3, or v5; see Fig. 3a). gj = Goły Jon; ol = Olsztyn; zmig = Żmigród; elk = Ełk; jul = Julianka. Manual inspections of gj_R263_v1, gj_R264_v1, ol_R152_v3, ol_R149_v5, and ol_R150_v3 revealed that they are true recombinants between v2 and v1 (gj) or v2 and v3/v5 (ol).

**Figure 2:**
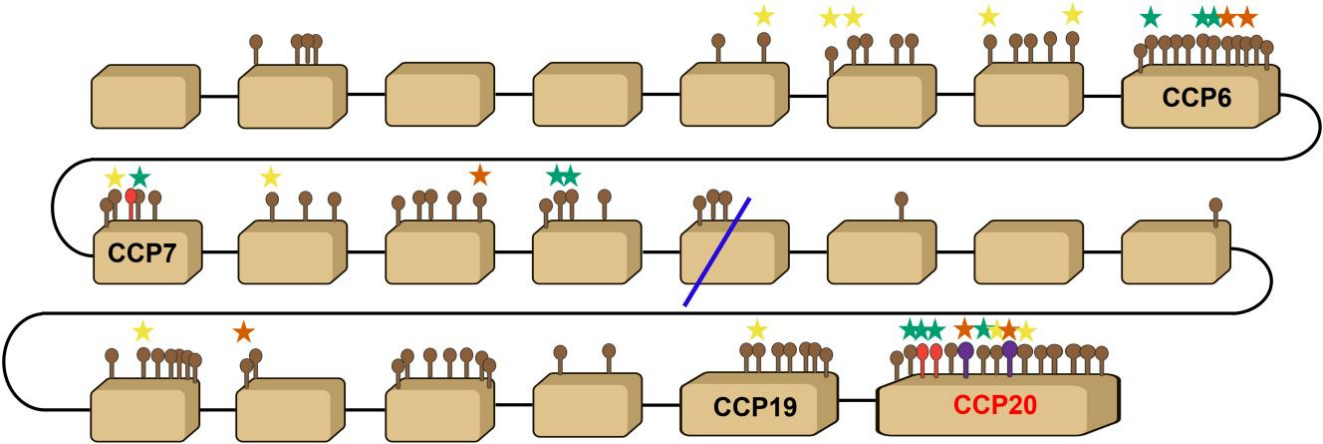
The CFH gene in bank voles presents many non-synonymous SNPs, particularly at CCP 6 and 20. Schematic representation of the full CFH gene. Brown boxes represent each of the 22 exons; exons corresponding to CCP 6, 7, 19, and 20 are labeled. Lollipops above boxes represent non-synonymous SNPs in bank voles and show their approximate location in the exon. Brown lollipops = non-synonymous SNP; red lollipops = non-synonymous SNP at a site involved in interactions with *Borrelia* or other pathogens (Meri et al. 2013; Cagliani et al. 2016); purple lollipops = non-synonymous SNP at a site involved in interactions with *Borrelia* and with the host's polyanions (Meri et al. 2013). Blue line = location of the split between the 2 partitions of the multiple sequence alignment. The Figure also shows sites under positive selection identified with PAML (red-orange stars), MEME (yellow stars), or both (green stars) that are also non-synonymous polymorphisms in bank voles. Sites under positive selection in PAML are those with a posterior probability of ω > 1 greater than 95% using the Bayes Empirical Bayes analysis from the M8 model. Sites under positive selection in MEME are those with *P*-value <0.05.

Amplification and ONT sequencing of full-length transcripts from 19 individuals revealed 22 unique full-length CFH sequences, with a predicted CDS length (3,748-3,769 bp) congruent with the well-annotated gene in *Rattus norvegicus* (3,708 bp; NM_130409.2) and *Mus musculus* (3,702 bp; BC066092-1). The analysis of CFH sequences may be challenging because of the presence of similar paralogs (CFH-related genes) and pseudogenes. Therefore, we further confirmed that the sequences represented a single gene by aligning them to a database of expressed CFH obtained by amplifying cDNA of CCP 17-20 from 50 individuals (*see Methods*). CFH-related genes lack CCP 17 and/or 18; thus, our cDNA sequences were indeed CFH (Pouw et al. 2015). Phylogenetic analysis of full-length CFH revealed 3 major clades (Fig. 1). The average sequence divergence between all variants was 0.014. The most polymorphic domain was CCP 20 (189 bp), containing 20 SNPs, 15 of which were non-synonymous (Fig. 2). A re-analysis of data from Niedziałkowska et al. (2023), following the method of Nei and Gojobori (1986) to calculate divergence, showed that CFH is among the 1.47% most diverged transcripts for non-synonymous SNPs and 0.96% for synonymous SNPs between 2 populations in NE and Central Poland.

We used PAML to identify sites under pervasive positive selection and MEME to detect sites under episodic positive selection across the rodent phylogeny. For both analyses, we used one randomly selected CFH sequence from each of the 3 major bank vole clades (labeled v1, v2, and v5 based on homology of the last exon with CCP 20 variants, *see below*), 11 Cricetidae species, 7 murids, and 1 spalacid as an outgroup (Figure S2). The multiple-sequence alignment (MSA) was divided into 2 partitions due to a potential recombination break point identified with GARD, and selection analyses were conducted independently for both partitions. The analysis with PAML revealed the presence of sites under positive selection in both partitions of the MSA, as the M2a and M8 models were better fits of the data than the nearly neutral models (Table S1). Specifically, PAML identified 32 sites in partition 1 and 11 sites in partition 2 with evidence of pervasive positive selection across the rodent phylogeny (Table 1). MEME identified 41 sites in partition 1 and 51 sites in partition 2 with evidence of episodic positive selection (Tables 2 and 3). Only 14 and 7 sites were identified by both methods for partitions 1 and 2, respectively. Most of the sites under positive selection within partition 2 lay in the CCP 20 domain (PAML: 7 sites, 63.6%; MEME: 11 sites, 21.6%; common sites: 5; Figure S3). Many of these positively selected sites within CCP 20 (namely E566, N577, M581, P584, R587, G591, and V610) overlapped or were directly adjacent to sites inferred to be involved in interactions with *Borrelia* (OspE and CspA), other pathogens, and/or self-components (Kraiczy et al. 2004; Meri et al. 2013; Cagliani et al. 2016). The 37.2% and the 22.8% of all the sites identified by PAML and MEME, respectively, harbor non-synonymous polymorphisms in bank voles and thus could potentially be under positive selection in this species (Fig. 2).

**Table 1:** Sites under positive selection identified by PAML in partitions 1 (left) and 2 (right). Sites 566 - 591 in partition 2 are within CCP 20.

Site	Pr(ω > 1)	ω posterior mean	SE	Site	Pr(ω > 1)	ω posterior mean	SE
**Partition 1:**				**Partition 2:**			
214	0.993	2.515	0.206	126	0.959	2.666	0.552
223	0.99	2.51	0.224	317	0.987	2.719	0.474
296	0.998	2.522	0.179	371	0.966	2.68	0.533
337	0.997	2.522	0.179	386	0.985	2.716	0.478
340	0.98	2.491	0.266	566	0.963	2.674	0.544
341	0.997	2.521	0.183	577	0.986	2.717	0.475
352	1	2.527	0.161	581	1	2.742	0.428
354	0.998	2.523	0.175	584	0.989	2.723	0.465
356	0.986	2.503	0.244	587	0.999	2.741	0.431
359	1	2.526	0.162	588	1	2.742	0.428
362	0.989	2.508	0.231	591	0.999	2.741	0.431
367	1	2.526	0.162	…	…	…	…
368	0.991	2.511	0.22	…	…	…	…
373	0.981	2.491	0.261	…	…	…	…
390	0.998	2.523	0.179	…	…	…	…
398	0.996	2.519	0.192	…	…	…	…
402	0.993	2.515	0.208	…	…	…	…
404	0.992	2.513	0.215	…	…	…	…
406	1	2.526	0.162	…	…	…	…
408	0.975	2.482	0.29	…	…	…	…
416	0.993	2.515	0.207	…	…	…	…
419	0.994	2.516	0.202	…	…	…	…
451	0.973	2.48	0.301	…	…	…	…
466	0.995	2.518	0.195	…	…	…	…
519	0.995	2.519	0.194	…	…	…	…
556	0.966	2.467	0.327	…	…	…	…
576	0.957	2.453	0.354	…	…	…	…
577	0.968	2.472	0.32	…	…	…	…
578	0.982	2.494	0.261	…	…	…	…
580	1	2.526	0.164	…	…	…	…
582	0.98	2.492	0.272	…	…	…	…
630	0.995	2.518	0.195	…	…	…	…

**Table 2:** Sites under episodic positive selection identified by MEME in partition 1. Alpha = synonymous substitution rate; beta + = non-synonymous substitution rate for the positive/neutral evolution component; *P* + = proportion of the tree evolving neutrally or under positive selection; LRT = likelihood ratio test for *P* + >0; # branches = number of branches under selection.

codon	alpha	beta +	*P* +	LRT	*P*-value	# branches
269	0.106	108.043	0.06	16.405	0.0001	2
354	0.665	65.785	0.244	15.829	0.0002	3
400	0	25.244	0.216	14.608	0.0003	3
402	0	28.132	0.217	13.441	0.0005	4
371	0	20.708	0.122	11.932	0.0011	3
359	2.335	505.736	0.17	9.608	0.0036	1
187	0.156	6382.443	0.028	9.355	0.0041	1
293	0	543.651	0.13	8.874	0.0052	1
226	0	24.999	0.071	8.849	0.0053	2
304	0	40.114	0.068	8.842	0.0053	2
102	0	24.343	0.069	8.455	0.0064	2
352	1.182	43.629	0.348	8.071	0.0078	0
431	0	12.381	0.089	7.84	0.0088	2
452	0	20.802	0.285	7.816	0.0089	2
393	0	90.013	0.116	7.471	0.0106	4
465	0	11.272	0.193	7.446	0.0108	3
49	0.164	245.28	0.05	7.292	0.0116	2
209	0.356	17.023	0.197	7.175	0.0124	4
578	0	3.058	1	6.975	0.0137	3
492	0	15.394	0.169	6.962	0.0138	3
337	0	11.54	0.488	6.952	0.0139	1
577	1.019	1127.726	0.031	6.94	0.0139	1
419	0	3.181	1	6.924	0.014	1
43	0	12.664	0.083	6.617	0.0164	2
214	0	5.263	0.716	6.407	0.0183	0
356	0.616	10.222	0.397	6.161	0.0208	3
341	0.514	4.081	1	6.039	0.0221	0
242	0.77	327.134	0.053	5.945	0.0232	1
218	0.577	65.699	0.041	5.877	0.024	1
211	3.286	10000.000…	0.038	5.696	0.0264	1
406	0	11.492	0.528	5.667	0.0268	4
76	0	45.4	0.056	5.203	0.034	1
404	0	7.393	0.544	5.195	0.0341	1
386	0	1.148	1	5.062	0.0365	3
273	0	5.657	0.308	5.03	0.0371	1
317	0	2.237	0.913	5.022	0.0373	2
74	0.545	9.602	0.188	4.686	0.0443	2
390	0	13.866	0.475	4.672	0.0447	2
276	0.548	8.688	0.308	4.591	0.0466	1
250	0	1.088	1	4.562	0.0473	1
45	0.801	20.223	0.037	4.473	0.0495	1

**Table 3:** Sites under episodic positive selection identified by MEME in partition 2. Alpha = synonymous substitution rate; beta + = non-synonymous substitution rate for the positive/neutral evolution component; *P* + = proportion of the tree evolving neutrally or under positive selection; LRT = likelihood ratio test for *P* + >0; # branches = number of branches under selection. Codons 450 - 610 are within CCP 20.

codon	alpha	beta +	*P* +	LRT	*P*-value	# branches
6	0.52	9999.999	0.047	4.582	0.0468	1
14	1.18	175.57	0.028	8.815	0.0054	1
16	0.167	643.907	0.029	5.41	0.0305	1
61	0.598	84.733	0.045	5.013	0.0375	1
98	1.714	55.412	0.078	4.797	0.0419	0
99	0	19.974	0.045	7.482	0.0106	1
103	0	8.448	0.202	5.63	0.0273	2
125	0.491	10000.000…	0.025	4.893	0.0398	1
126	1.302	906.639	0.084	6.914	0.0141	0
137	0	8.397	0.116	5.439	0.0301	2
156	1.669	51.703	0.072	4.591	0.0466	1
158	0.589	12.108	0.211	5.771	0.0254	3
176	0.682	157.567	0.06	8.85	0.0053	2
178	0	8.693	0.326	4.889	0.0399	2
211	0	11.453	0.044	4.611	0.0461	1
241	0	2.439	0.663	4.526	0.0482	1
249	0.167	281.881	0.054	18.63	0	2
259	0.915	24.099	0.102	7.464	0.0107	3
264	0	41.547	0.062	15.486	0.0002	2
299	0	4.944	0.443	5.855	0.0243	1
325	0	9.304	0.176	7.004	0.0135	3
346	1.301	50.121	0.059	6.714	0.0156	2
352	0.154	1083.305	0.031	7.068	0.0131	1
357	0	29.083	0.066	7.886	0.0086	2
366	0	8.177	0.165	5.234	0.0334	3
371	0.6	41.189	0.181	6.794	0.015	1
381	0	2.661	1	6.262	0.0197	1
415	0.419	9.2	0.176	5.392	0.0308	2
429	0	1.213	1	4.636	0.0455	1
445	0	9.638	0.286	8	0.0081	1
450	0	10.225	0.144	6.411	0.0183	1
454	0.419	1175.682	0.031	7.592	0.01	1
467	0.121	55.289	0.041	12.503	0.0008	1
469	0	916.119	0.026	12.405	0.0009	1
470	0.779	385.265	0.026	6.807	0.0149	1
472	1.419	41.524	0.03	4.898	0.0397	1
474	0	352.462	0.026	7.258	0.0119	1
514	0	27.502	0.079	7.449	0.0108	2
521	0.922	10.096	0.245	5.982	0.0228	5
522	0.719	79.375	0.121	11.484	0.0014	2
566	0	7.409	0.478	6.221	0.0201	5
572	1.196	359.645	0.026	4.47	0.0496	1
575	1.851	243.261	0.026	5.689	0.0264	1
577	0	14.579	0.432	7.887	0.0086	2
581	1.407	97.869	0.38	8.149	0.0075	1
584	0	4.091	0.855	7.954	0.0083	2
588	1.294	11.354	1	5.773	0.0253	0
589	0	17.815	0.356	11.124	0.0017	4
598	0	1.721	1	4.709	0.0438	0
608	0	6.109	0.23	4.572	0.047	3
610	0.545	18.927	0.27	4.885	0.04	0

In addition to CCP 20, PAML and MEME identified many more codons under positive selection in CCP 6 (11 by PAML and 8 by MEME) and in CCP 7 (where both methods detected 8 sites) compared to other domains (Figure S4). Five and 4 sites under positive selection in CCP 7, identified by PAML and MEME, respectively, were within a region bound by *Borrelia*'s CspA (Kraiczy et al. 2004).

The analysis with aBSREL showed that the bank vole lineage was under positive selection, as the branch leading to bank voles in the rodent phylogeny was predicted to be subject to positive selection within both MSA partitions (partition 1: rates = 2, max ω = 19.04 in 7.92% of sites, *P*-value ≪0.001; partition 2: rates = 2, max ω > 1000 in 2.2% of sites, *P*-value = 0.001; Tables S2 and S3). Additionally, the branch leading to the bank vole variant v2 was under positive selection in partition 1 (rates = 2, max ω > 1000 in 0.19% of sites, *P*-value = 0.012), whereas the branch leading to variant v1 was under positive selection in partition 2 (rates = 2, max ω = 43.76 in 5.88% of sites, *P*-value = 0.006) of the MSA.

Taken together, the results show that CFH in bank voles is highly polymorphic and evolved under positive selection. Across the rodent phylogeny, the sites subject to positive selection include amino acids directly involved in interactions with pathogens.

### CCP 20 diversity shows a geographical pattern

**Figure 3:**
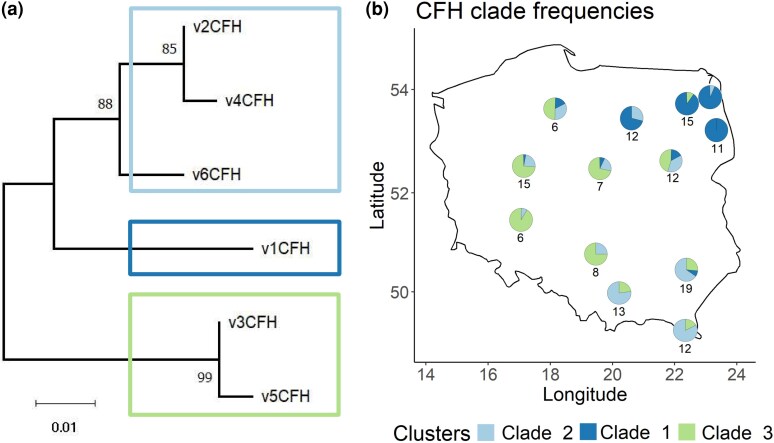
CFH is highly polymorphic with variants clustering in 3 clades with different frequencies across populations. (a) Neighbor-joining phylogenetic tree of the CFH variants at the CCP 20 domain. Numbers at nodes show bootstrap values. (b) Map of Poland with overlaid pie charts showing CFH clade frequencies per population. Numbers under (or above) the pie charts are sample sizes per population (number of sequenced individuals). Azure = clade 2 (v2, v4, and v6); blue = clade 1 (v1); green = clade 3 (v3 and v5). The same individuals as for RAD-seq were sequenced; however, CFH amplification failed for a few individuals.

We then focused on the CCP 20 domain, as this is the most polymorphic region and is functionally important for interactions with pathogens and self-molecules. We first amplified the fragment of 189 bp covering CCP 20 and then filtered co-amplifying CFH-related genes and possible pseudogenes by mapping sequences to the database of cDNA CCP 17-20 (*see above*). We found a total of 6 unique CCP 20 variants in our sample of 143 voles. All genotyped individuals had one or 2 CCP 20 CFH variants, confirming their allelic status. The phylogenetic analysis of the 6 CCP 20 alleles revealed 3 main clades corresponding to the main clades identified with the full sequence tree (Figs 1 and  3a). The clades show a clear geographical pattern (Fig. 3b). Clade 2 consists of 3 variants (v2, v4, and v6) and dominates in the SE, clade 1 contains only one variant (v1) and dominates in the NE, while clade 3, comprising 2 variants (v3 and v5), dominates in the SW. Populations from central regions show mixed frequencies of all clades.

### CFH shows a stronger population structure than the genomic background

**Figure 4:**
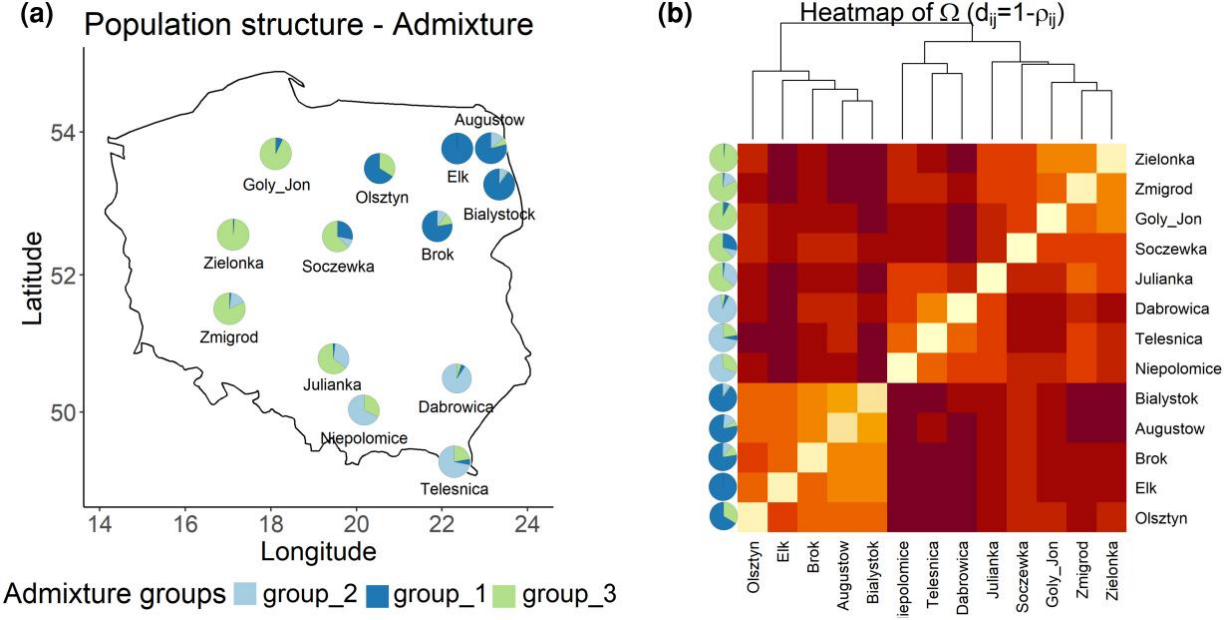
Bank vole populations in Poland cluster into 3 main genomic groups. (a) Population structure identified with Admixture analysis of RAD-seq SNPs (K = 3). Pie charts show the admixture ancestry proportions (Q) averaged by each population. Azure: = group 2 (∼Southern clade); blue = group 1 (∼Eastern clade); green = group 3 (∼Carpathian clade). Given that the 10 admixture runs with different seeds gave comparable results, Q values from one run chosen at random are shown. (b) Heatmap showing the population structure identified in the covariance (Ω) matrix. The lighter the color of the cell, the stronger the correlation between the 2 populations. The Ω matrix was scaled to a correlation (similarity) matrix (ρ_ij_), and the dissimilarity matrix (d_ij_) between populations (i and j) was plotted. The tree was generated with hierarchical clustering using the average agglomeration method. The pies on the left are the same as in (a).

Next, we compared the geographical pattern identified in CCP 20 with the overall bank vole population structure. To this aim, we conducted RAD sequencing (BioProject PRJNA1366785) on the same individuals screened for CCP 20 to obtain a reduced representation of the genome. SNP calling and genotyping of the RAD-seq data using Stacks resulted in 58,535 markers after filtering and thinning of closely positioned SNPs. Overall, all populations showed low differentiation (average pairwise *F*_ST_ = 0.032 ± 0.027 SE; Figure S5) and had a mean nucleotide diversity (π) = 0.23-0.25 (Table S4). Admixture analysis of population structure using RAD-seq markers revealed that populations cluster into 3 main groups (*K* = 3 had the lowest cross-validation error; Figure S6), and their structure is congruent with the geographical pattern observed in CCP 20 (Figs 3b and  4a). Central populations show larger degrees of admixture, consistent with their geographical locations (Fig. 4a).

We then statistically assessed the congruence between genomic population structure and the geographical pattern of the CCP 20 variants, testing whether structure is stronger in CCP 20 compared to the genomic average. To do so, we conducted genomic scans of selection using the hierarchical, Bayesian method implemented in BayPass (Gautier 2015). This is a robust outlier test that accounts for complex demographic histories, such as the one found in European bank vole populations that expanded from multiple glacial refugia (Wójcik et al. 2010; Marková et al. 2020). This method, therefore, does not make a priori assumptions on the demographic history of populations, which is instead incorporated in a covariance matrix (Ω) estimated from allele frequencies and reflecting the neutral correlation structure among populations. Genomic scans were conducted using the RAD-seq markers and the SNPs in CCP 20. Of the 20 SNPs in this domain, 6 were perfectly linked (they were adjacent and occurred in the same variants); therefore, they were clumped together in the analyses, which were therefore conducted on 16 variants.

**Figure 5:**
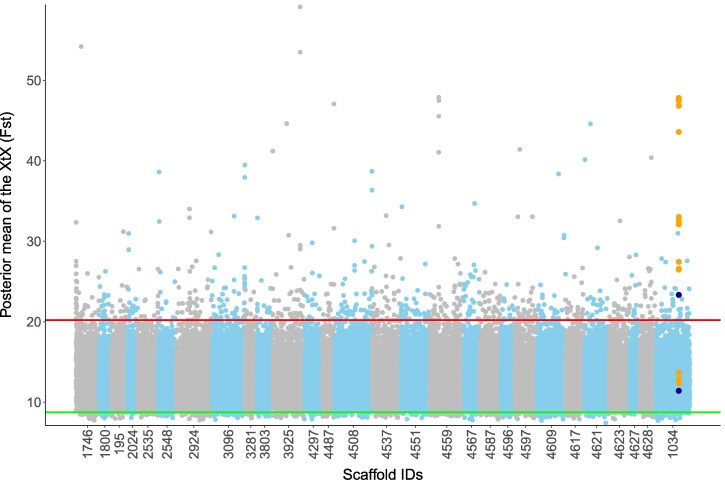
CFH SNPs are differentiated among populations and under diversifying selection. Manhattan plot showing the results of the genomic scans for signatures of selection. X-axis: cumulative genomic position in base pairs. The labels are the truncated scaffold IDs (the part after the “HRSCAF=” in the labeling system for scaffolds used in the reference genome [Marková et al. 2023]); for graphical reasons, labels for the scaffolds with less than 2 SNPs are not shown, and their SNPs were combined with other scaffolds. Y-axis: mean from the posterior distribution of the X^T^X for each SNP. Each dot is a RAD-seq SNP; orange dots = SNPs in the CCP 20 domain; dark blue dots = the 2 RAD-seq SNPs inside the CFH gene. Scaffolds are shown with alternating colors. Red line = 99% quantile from the POD's X^T^X null distribution; green line = 1% quantile from the same distribution.

The structure identified in the Ω matrix was congruent with that from Admixture (Fig. 4b). The genomic scans revealed that 13 of the 16 SNPs in the CCP 20 domain were outliers, showing stronger differentiation than expected from genome-wide data (Fig. 5). Additionally, the RAD-seq dataset contained 2 SNPs located in intronic regions inside the CFH gene, of which one was found to be an outlier under diversifying selection. Therefore, CFH and, particularly, the CCP 20 domain are more structured than the genomic average.

### Outlier RAD markers are enriched for innate immunity and the alternative complement pathway

In addition to CFH, we identified 758 and 505 RAD SNPs showing extremely high or low differentiation, suggesting diversifying or balancing selection, respectively. We linked these to their closest genes and thus identified 271 and 195 unique, annotated genes under diversifying or balancing selection, respectively. The remaining outlier SNPs overlapped with, or were in proximity to, genes without annotations or located within transposable elements.

Enrichment tests on the genes linked to RAD-seq SNPs under diversifying selection revealed 11 significantly enriched terms (Dataset S1; Figure S7a), including 2 immunity-related categories: the “complement activation, alternative pathway” term (GO:0006957), to which CFH belongs, and “innate immune response” (GO:0045087). The 3 outlier genes belonging to the first term were CFH, C3, and C5 (Dataset S1). Among the terms enriched for the SNPs under balancing selection, we found 12 significant terms (Dataset S2; Figure S7b), including “positive regulation of interleukin-6 production” (GO:0032755) and “interleukin-6-mediated signalling pathway” (GO:0070102). Overall, in addition to the CCP 20 of CFH, other components of the alternative complement pathway, are under diversifying selection.

## Discussion

CFH is a key regulatory protein of the alternative complement pathway that is often hijacked by pathogens. Here, we describe a naturally occurring polymorphism in this gene and investigate molecular and population genetics signatures of selection acting on it in natural populations of the bank vole, one of the key European reservoirs of zoonotic pathogens, including *Borrelia afzelii* (Hanincová et al. 2003; Grzybek et al. 2020). We show extensive CFH polymorphism, particularly within domain 20 (CCP 20), involved in the protection of self-cells and serving as a binding site for pathogens, allowing immune evasion. Furthermore, this polymorphism is associated with signatures of natural selection in terms of (i) an excess of non-synonymous substitutions at several codons and (ii) elevated inter-population differentiation compared to the genome-wide average. Moreover, using RAD-seq, we identified additional outlier SNPs under diversifying selection in the proximity of innate immunity genes, including those involved in the alternative complement pathway.

### Polymorphism and selection on CFH

Polymorphism, coupled with signatures of positive selection, appears to be a common feature of genes involved in parasite recognition, including MHC genes (Hughes and Nei 1988; Bernatchez and Landry 2003) and Toll-like receptors (TLRs; Minias and Vinkler 2022), likely due to cycles of adaptations and counter-adaptations with pathogens (Bodmer 1972; Ejsmond and Radwan 2015; Migalska et al. 2022). However, whether similar mechanisms also apply to the regulators of missing-self immunity, which instead recognize molecular markers of self (Carrillo-Bustamante et al. 2016) and may therefore be evolutionarily constrained, is not as well understood.

Here, we found evidence of high polymorphism in CFH, one such regulator of missing-self immunity, confirming and extending results from Niedziałkowska et al. (2023), where 2 variants were identified in de novo transcriptomes from 2 populations. One explanation for this polymorphism is that CFH is under selective pressure resulting from immune evasion by pathogens. Our analyses identified signals of positive selection at sites in the CCP 20 domain adjacent to or overlapping with those involved in interactions with pathogen proteins. While these codons were identified in the whole rodent phylogeny, they harbor non-synonymous polymorphisms in bank voles, whose branch evolved under positive selection, suggesting that strong selective pressures to prevent pathogens from binding are likely acting on CFH in this species. Our results are remarkably similar to those reported for interspecific analyses of primate CFH (Cagliani et al. 2016), where signals of positive selection were detected in CCP 20 and in the *Borrelia* OspE sites interacting with CFH. Positive selection in OspE, in particular, lends further support to the host–parasite coevolution hypothesis. Overall, these results suggest that selection in response to immune evasion is pervasive among mammals and that different sites within this domain may affect the binding of pathogen proteins. To the best of our knowledge, a similar coevolutionary scenario was identified in only one other regulator of missing-self immunity before our study, the killer-cell receptor (Carrillo-Bustamante et al. 2015, 2016).

The apparently adaptive polymorphism at CFH, particularly at CCP 20, is remarkable considering that this domain (along with CCP 7) is involved in binding to self-signals for immune recognition (Meri 2016). This could possibly result in trade-offs between efficiency of binding to self-signals and avoidance of immune evasion. A recent theoretical study (Hummert et al. 2018) explored the role of such a trade-off in the maintenance of polymorphism at CCP 7, known in humans to be related to autoimmunity. The modeling showed that the trade-off may lead to the maintenance of stable polymorphism within populations if heterozygotes best balance infection resistance against autoimmunity. Whether the CCP 20 polymorphism leads to autoimmunity in bank voles deserves further investigation; however, we note that our results show higher population structuring at CFH compared to the genomic average, which is contrary to the predictions from heterozygote advantage, where the opposite pattern would be expected (Beaumont and Balding 2004).

The distribution of the CFH clades corresponded to the 3 genetic groups that we detected with RAD markers, suggesting both have been shaped by phylogeographic history. In addition to the 2 CFH divergent haplotypes inferred from transcriptomes by Niedziałkowska et al. (2023), which dominate respectively in NE (clade 1) and SW (clade 3), we detected a third, previously undescribed CFH clade comprising the highest number of variants (clade 2) that dominated in SE. The 3 genomic groups, also identified by RAD-seq analysis in Niedziałkowska et al. (2023), although on a more limited geographic scale, likely reflect the history of postglacial colonization (Kotlik et al. 2006; Wójcik et al. 2010; Marková et al. 2020). However, divergence of CFH major lineages is much higher than the genomic average. Indeed, the net synonymous divergence between our 2 most diverged bank vole CFH clades (clade 1 and 3) is 0.0184, which is about 4.6% of the average synonymous divergence between bank voles and yellow-necked mice immune genes (i.e. ∼0.4; Zhong et al. 2021). Since the 2 species split 26.2 Mya (according to TimeTree.org; Kumar et al. 2022), we estimate that CFH accumulated divergence for about 1.2 My. This indicates that CFH polymorphism in bank voles predates the LGM estimated to have occurred 18 to 23 kya (Mix et al. 2001).

The spatial patterning of CFH, consistent with genome-wide patterns, might have arisen via 3 non-exclusive scenarios. According to the first scenario, CFH could have diverged via selection throughout the Pleistocene, leading to local adaptation during glacial periods with no or little mixing during interglacial periods; however, it is not clear how to reconcile this scenario with the lower divergence of bank vole mitochondrial clades (30 to 70 kya; Wójcik et al. 2010), which is estimated to have occurred only during the last glaciation (ca. 115 to 11.7 kya). Alternatively, several CFH allelic lineages may have been maintained for a long time by balancing selection and then sorted differently in the glacial refugia. Extreme synonymous divergence of bank vole CFH clades compared to other expressed genes does indeed suggest long-term maintenance of the CFH lineages. While the higher CFH allele frequency differentiation compared to the genomic average does not support heterozygote advantage, balancing selection may have occurred via negative frequency-dependent selection (NFDS). NFDS may lead to rapid fluctuations in allele frequencies (e.g. Westerdahl et al. 2004; Migalska et al. 2022), and if host-parasite allele frequency cycles are out of phase in populations with limited gene flow, this can lead to higher population differentiation compared to neutral expectations, mimicking the signal of local adaptation (Spurgin and Richardson 2010). Finally, in the third scenario, the geographic pattern of CFH would be due to admixture from an unknown source. Overall, our results indicate that selective forces, likely stemming from adaptation to pathogens, contribute to the inter-population differences in CFH allele frequencies. Whether selection on CFH is currently ongoing, possibly causing alleles to flow at a lower rate compared to the genomic average, deserves further investigation.

### Outliers at RAD-associated immunity genes

While our focus was on CFH, our analysis also identified several outliers in the RAD data (based on the X^T^X metric, an analogue of F_ST_ that accounts for the variance-covariance structure of the populations; Günther and Coop 2013) enriched for various GO terms. Discussing all these outliers in detail is outside the scope of this study; here, we briefly discuss the outlying immunity genes. One of the GO terms that showed significant enrichment was “complement activation, alternative pathway”, which includes C3 and C5. C3 is a complex protein consisting of several subunits, each with different functions (Geisbrecht et al. 2022; Zarantonello et al. 2023). One subunit, C3b, binds to PAMPs to induce opsonization and further complement activation; the same subunit is also a target of immune evasion by *Staphylococcus aureus* in humans (Garcia et al. 2010; Geisbrecht et al. 2022). Additionally, mutations in C3 were linked to autoimmune disease (Geisbrecht et al. 2022; Zarantonello et al. 2023); therefore, population differentiation at this gene may result from a complex interplay of opposing selective pressures, ie increased binding efficiency to PAMPs, escaping binding from pathogens, and evolutionary constraints due to autoimmune disease. C5 is a downstream component of complement that is also the target of immune evasion from pathogens from different taxa (bacteria, ticks, fungi; Laursen et al. 2012; Luo et al. 2013). Both CFH and C5 interact with C3, and the specificity of their interaction may contribute to population differentiation. Enrichment of complement cascade genes among those subject to local adaptation contrasts with findings from Nandakumar et al. (2023). This study reports significant signatures of balancing selection acting on 4 of the 44 complement cascade genes investigated, based on β population statistics reflecting a build-up of alleles at similar frequencies. These 4 genes did not include those we found to be subject to diversifying selection, so our data highlight that different components of the complement system may be subject to different coevolutionary dynamics. This is not unexpected given that host-parasite coevolution can take the shape of both balancing selection and recurrent selective sweeps (Woolhouse et al. 2002).

Additional immune genes closest to the SNPs with evidence for diversifying selection in our study include CD180 antigen, Annexin A1 (ANXA1), Ankyrin repeat domain 17 (ANKRD17), interferon-induced protein with tetratricopeptide repeats 1 (IFIT1), and interleukin 1 receptor accessory protein (IL1RAP). CD180 (a Toll-like receptor) and IFIT1 bind to PAMPs (McDougal et al. 2024; Xie et al. 2025), with IFIT1 also being a target for immune evasion (Xie et al. 2025). Thus, because of their function, these genes represent potential candidates for host–pathogen coevolution. Polymorphisms in IL1RAP, ANKRD17, and ANXA1 are associated with disease (Vilkeviciute et al. 2020; Yu et al. 2020; Granell et al. 2023; Tinatin et al. 2023). These genes are involved in immunity as signal transducers, interacting with hosts' receptors, with no evidence so far that they could be targets of immune evasion. Immunity genes were also represented among the outlier loci under balancing selection, including enrichment of loci involved in the interleukin 6 signaling pathways. Interestingly, among the genes in this pathway, we found TLR6, which was the only TLR without evidence of balancing selection (based on Fu and Li's D* and Tajima's D) in the bank vole populations investigated in Kloch et al. (2018). The reasons for the differences between these findings require further investigation.

## Conclusions

Our study revealed extensive polymorphism in CFH, particularly in CCP 20, which included several candidate sites under positive selection. Coevolution with pathogens whose surface proteins bind to this domain to evade destruction by the complement cascade seems the most likely explanation for this result, further supported by population genetic evidence for diversifying selection acting on CCP 20. Importantly, our findings provide novel evidence that genes involved in missing-self immunity, such as CFH, which are typically thought to be evolutionarily constrained due to their essential roles in self-recognition, can also be targets of adaptive evolution. The consequences of this naturally selected polymorphism on host fitness need to be addressed in future studies. More broadly, this work indicates that genes involved in innate immunity, particularly in the alternative complement pathway, are among the most differentiated across populations, likely reflecting selective pressures imposed by pathogens.

## Material and methods

### Sampling

To capture bank voles' representative diversity, sampling was conducted at 13 locations across Poland, spanning the contact zone between the 2 major phylogeographic lineages, Carpathian and Eastern, as identified by mitochondrial DNA (Wójcik et al. 2010; Marková et al. 2020; Table S5; Figure S8). We collected ear biopsies from *C. glareolus* in 2020-2023 during August-September under approval of the Local Ethics Committee for Animal Experimentation in Poznań, decision no. 35/2021. The samples were promptly stored in ethanol and frozen until DNA extraction. A subsample was preserved in RNAlater Stabilization Solution (Thermo Fisher Scientific, Waltham, MA, USA) for RNA extraction. Samples from Teleśnica and Niepołomice were provided by Prof. Paweł Koteja (Jagiellonian University, Kraków, Poland) and were collected in 2009 (under approval of the local Ethics Committee in Kraków, Decision No. 48/2007). DNA was extracted from bank vole ear samples with the NucleoMag 96 Tissue Kit (Macherey-Nagel, Duren, Germany) following the manufacturer's protocol. Total RNA was extracted with the ReliaPrep RNA Tissue Miniprep System (Promega, Madison, WI, USA). cDNA was synthesized using Maxima H Minus First Strand cDNA synthesis Kit (Thermo Fisher Scientific, Waltham, MA, USA).

### CFH genotyping-by-sequencing

To obtain full-length CFH sequences, we used primers CCP0-F: 5′- ATGAGACTATCAGCAAGAATTATCTGGCT-3′ and CCP20-R: 5′- CATGAATATTTCTCAACAAAKRTTCA-3′, amplifying cDNA of a total length of 3,780 bp spanning exons 1 to 22. These primers were designed based on de novo assembled transcripts from Niedziałkowska et al. (2023) and Różańska-Wróbel et al. (2025) aligned with *M. musculus* transcripts (NCBI Reference Sequence: NM_009888.3) for exon identification. We used these primers to amplify CFH sequences from the cDNA of 19 individuals from 9 populations across Poland (Table S6), which were then sequenced using ONT MinION with the V14 rapid barcoding kit. Consensus sequences were obtained with the SLANG pipeline (Dorfner et al. 2022), which uses VSEARCH (Rognes et al. 2016) to cluster reads within and then between samples based on similarity thresholds. Since we wanted to identify different alleles from a single locus, we used very stringent similarity thresholds, ie 0.97 and 0.99 for within- and between-sample clustering, respectively. All other settings were set to default. We selected and manually inspected the CFH consensus sequences with a length >3,400 bp and supported by the largest number of reads.

We then focused on the most polymorphic domain of CFH, CCP 20. Based on full-length sequences, we designed primers 20F and 20R (CCP20-F: 5′-CATGTGTAATATCAGAAGAGAYCA-3′ and CCP20-R: 5′-CATGAATATTTCTMAACAAAGGTTCAT-3′) to amplify CCP 20 from genomic DNA. The primers were used to amplify CFH CCP 20 from the same samples as for RAD-seq; however, 11 amplifications were unsuccessful. The primers were barcoded to identify unique samples as described by Kozich et al. (2013), except that the “N” (0 to 3 nucleotide) spacers were added to increase library diversity during Illumina sequencing. PCR was performed using the Type-it Microsatellite PCR Kit according to the manufacturer's protocol. The reaction setup contained 1 µL of template DNA (∼100 ng), 1 µL of CCP20-F primer (final concentration: 0.4 µM), 1 µL of CCP20-R primer (final concentration: 0.4 µM), 12.5 µL of 2x Type-it Multiplex PCR Master Mix, and nuclease-free water up to 25 µL. Thermal cycling was performed with an initial denaturation at 95 °C for 5 min, followed by 28 cycles of denaturation at 95 °C for 30 s, annealing at 58 °C for 90 s, and extension at 72 °C for 30 s, with a final extension at 60 °C for 30 min. CFH amplicons were sequenced using the Illumina MiSeq platform with the MiSeq Reagent Micro Kit v2 (paired-end, 2 × 150 bp). The resulting reads were merged using AmpliMERGE and analyzed with AmpliSAS for demultiplexing, clustering, and filtering of unique variants (Sebastian et al. 2016). We used default settings for Illumina reads and enabled the “discard frameshifts” option.

We obtained up to 11 variants per individual (instead of the expected two), suggesting that the CCP 20 primers, in addition to CFH, also amplified CFH-related genes and possibly pseudogenes. To exclude these non-CFH genes, we first created a database of expressed CFH, obtained by sequencing cDNA from a representative sample of 50 individuals from 9 populations (Table S7). Because CFH-related genes do not have CCP 17, and most of them also miss CCP 18 (Pouw et al. 2015), we amplified cDNA in a nested PCR using forward primers CCP17-F (5′-TGATTGTGACAGTTTACCCAAGTATGA-3′, 10 cycles) and CCP18-F (5′-TGATGTGTCAAAATGGGATTTG-3′, 20 cycles) and the reverse primer CCP20-R to selectively exclude CFH-related genes. The amplicons were sequenced using the Illumina MiSeq platform with the Reagent Kit v3 (paired-end, 2 × 300 bp). Since we expected to obtain sequences from a single expressed locus, we checked and confirmed that each individual possessed only one or 2 alleles. To exclude CFH-related genes and pseudogenes, we then mapped the CCP 20 sequences obtained from genomic DNA to the database of 7 expressed CFH sequences; matching CCP 20 sequences (6 variants) were retained for downstream analysis. All individuals in our sample had 1 or 2 variants of the filtered sequences, confirming their allelic status.

Additionally, we reanalyzed transcriptomic data originating from western (Włocławek) and eastern (Białowieża) Poland (Niedziałkowska et al. 2023). Raw reads were filtered using Trimmomatic (Bolger et al. 2014) and mapped to the reference transcriptome with BWA mem (Li, 2013). Duplicate reads were marked with samtools markdup, and reads with a mapping quality below 20 were removed. Genotypes were called across all sites using bcftools, and positions with a quality score or a mean mapping quality below 30 were excluded. For downstream analyses, we retained only genotypes with a minimum depth of 6. Using a custom Python script, we calculated synonymous and nonsynonymous divergence between the eastern and western populations following the method of Nei and Gojobori (1986). We also estimated within-population nucleotide diversity at synonymous and nonsynonymous sites, as well as net divergence. All analyses were performed per protein-coding gene, and the resulting values for the predicted CFH gene were compared against the genome-wide distribution obtained from all genes.

### Positive selection on CFH (d_N_/d_S_ analyses)

To identify signatures of positive selection, we conducted nonsynonymous-to-synonymous substitution (d_N_/d_S_) analyses on the full CFH transcripts of the 3 divergent bank vole variants, along with available sequences of other Cricetidae as well as Muridae. To obtain CFH sequences from related species, we used blastn to search for CFH in published Iso-seq databases from Muridae and Cricetidae (*Cricetulus griseus*: DRR568657-DRR568660; *Mus spicilegus*: ERR9929550-ERR9929561; *Mus spretus*: ERR9929562-ERR9929573; *Mesocricetus auratus*: SRR12589345, SRR14718534, SRR18449643; *Peromyscus maniculatus*: SRR29268902; *Microtus pennsylvanicus*: SRR30640723, SRR30640724). As a query, we used one of our full CFH sequences for all databases (“jul_R289_v5”) and the mouse CFH (BC066092-1) for Muridae databases. An e-value threshold was set at 1e^−30^. We then filtered the results to retain identified sequences with an alignment length greater than 3,000 bp; for each species and accession, we selected the sequence with the highest bitscore. Finally, sequences were extracted from fastq files with seqkit grep (Shen et al. 2016). Manual checks revealed a high degree of divergence within *M. spicilegus* and *M. spretus* (ie between their own accessions) that was comparable to divergence between species, suggesting extensive sequencing errors. This result was obtained both when using sequences blasted from bank vole or mouse CFH. We therefore discarded those species from the following analyses. For the species with multiple accessions, the sequence with the highest bitscore and length was chosen. We additionally blasted the “brok_R122_v2” and mouse sequences to the NCBI database using blastn and downloaded CDSs predicted from annotated genome assemblies of Cricetidae and Muridae. We only retained sequences with full CFH transcripts. When the same species had multiple hits, one sequence was randomly chosen, whereas for the species identified with both methods (Iso-seq and genomic predictions), the Iso-seq sequence was preferred. This resulted in the inclusion of 13 more sequences/species (*Meriones unguiculatus*: XM_060364402.1; *Chionomys nivalis*: XM_057769657.1; *Microtus ochrogaster*: XM_005348454.2; *Microtus oregoni*: XM_041647634.1; *Rattus rattus*: XM_032915223.1; *Arvicola amphibius*: XM_038348731.2; *Peromyscus eremicus*: XM_059280089.1; *Peromyscus californicus insignis*: XM_052737108.1; *Apodemus sylvaticus*: XM_052201275.1; *Arvicanthis niloticus*: XM_034512832.2; *Psammomys obesus*: XM_055624681.1; *Phodopus roborovskii*: XM_051183175.1; *Nannospalax galili*: XM_008831575.3). CFH CDSs for *M. musculus* (BC066092-1) and *R. norvegicus* (AJ320522.1) were directly downloaded. The resulting fasta file containing the 19 CFH orthologs from related species was complemented with 3 bank vole variants, one from each of the 3 clades (“jul_R289_v5”, “gj_R262_v1”, “brok_R122_v2”).

The sequences were first aligned using Macse (Ranwez et al. 2011), trimmed to the same length and excluding the stop codons; the quality of the alignment was visually checked with BioEdit (Hall 1999) to confirm the absence of obvious misalignments, frame shifts, gappy columns, and internal stop codons. The resulting MSA was then screened for the presence of recombination using GARD (Kosakovsky Pond et al. 2006) with a general time reversible (GTR) substitution model and a gamma-distributed site-to-site rate variation (this was identified as the DNA substitution model with the lowest BIC using MEGA11; Tamura et al. 2021). The GARD analysis provided model-support evidence for a potential recombination event at position 633 of the MSA and provided support for different tree topologies among the non-recombinant segments. Therefore, for the following selection analysis, we split the MSA into 2 partitions: positions 1-633 and 634-1,243.

The 2 MSAs were used to generate phylogenetic trees with MEGA11 using the maximum likelihood method tested with bootstraps (1,000 replications), the GTR DNA substitution model, and a gamma-distributed rate variation (Figure S2). We then conducted tests of d_N_/d_S_ with PAML (Yang 2007) and MEME (Murrell et al. 2012). We ran PAML under all site models (M0, M1a, M1b, M7, and M8) using a mutation-selection model with observed codon frequencies as estimates. Significant differences between nested models were checked using likelihood ratio tests, i.e. M0 vs M1a, M1a vs M2a, and M7 vs M8. We considered positively selected sites those with a posterior probability of ω > 1 greater than 0.95 using the Bayes Empirical Bayes analysis from the M8 model.

MEME was run with default settings, except that multiple site substitutions were estimated. We considered as sites with evidence of episodic positive selection those with a *P*-value <0.05.

We also used aBSREL (Smith et al. 2015) to test whether a proportion of sites within bank vole branches evolved under positive selection. We ran aBSREL with default settings and independently for the 2 MSA partitions, using as foreground branches the 3 bank vole variants, the branch leading to bank voles, and the internal branch within bank voles that separates 2 variants from the third.

The average sequence divergence between all full-length CFH sequences was calculated with the *nuc.div()* function from “pegas” (Paradis 2010), which uses Nei's (1987) formula.

### RAD sequencing protocol

To obtain a genomic background for variation and genetic structuring, the DNA from all samples genotyped for CCP 20 was sequenced with the 3RAD approach (Bayona-Vásquez et al. 2019), a reduced-representation library sequencing method. It is based on cutting DNA with a set of restriction enzymes, resulting in a common fraction of loci across samples, which allows for the identification of single-nucleotide polymorphism (SNP) markers. Libraries were constructed following the protocol provided in Bayona-Vásquez et al. (2019), with the following differences: (i) Illumina indexed primers were incorporated in a single PCR, with 12 cycles; (ii) purification at all steps was performed using AMPure XP magnetic beads (Life Sciences), following manufacturer's protocol; and (iii) selected size range was 300 to 600 bp. The size range of the DNA fragments for RAD sequencing was chosen based on the results of the *in-silico* digestion of the *C. glareolus* genome (accession: GCF_902806735.1) using the 3 enzymes of the 3RAD protocol (*XbaI*, *EcoRI*, and *NheI*) and a modified Python script from Driller et al. (2020). The results showed that the 300 to 600 bp size range would yield 20,000 to 30,000 loci for population genomic inferences (Figure S9).

Samples were divided into 3 pools, and a set of adapters including internal indexes (i5 and i7 adapters from Table S3 in Bayona-Vásquez et al. [2019]) was individually ligated to samples in each pool. Each one and a half pool received a different combination of Illumina indexes (iTru5_001_A-iTru7_101_01 and iTru5_001_B-iTru7_101_02 from Table S3 from Bayona-Vásquez et al. [2019]). Combining the half pools with the full pools containing the alternative combination of Illumina indexes allowed us to perform the paired-end sequencing (150 cycles) of all samples in 2 lanes of NovaSeq 6,000 SP flow cell (Illumina, San Diego, CA, USA). An addition of 20% PhiX was used to counterbalance the effect of fixed enzyme restriction sites. The raw (demultiplexed and adapter-trimmed) RAD-seq reads were made available on NCBI (BioProject PRJNA1366785).

### SNP calling and genotyping

RAD-seq data processing and loci building and genotyping were conducted with the Stacks pipeline (Catchen et al. 2013; Rochette and Catchen 2017) and other tools operating in R v.4.2.2 (R Core Team 2022). Firstly, we used the process_radtags program in Stacks to demultiplex the raw reads from the Illumina sequencing run (barcodes in line with sequence on both paired-end reads), remove any read with an uncalled base, discard reads with average quality scores (phred) within any sliding window <10 (window size = 15% of the length of the read), truncate reads to a length of 140 bp, and filter the Illumina adapters with 1 mismatch allowed in the adapter sequence. We assessed the quality of each sequenced sample and generated summary reports using the fastqcr package (Andrews et al. 2010; Kassambara 2023). Samples with notably low coverage, containing fewer reads than 10% of the median of all samples (302,396.5 reads), were dropped (65 samples; Figure S10).

Following Paris et al. (2017), before running the Stacks pipeline, we conducted optimizations of the 3 Stacks parameters that define initial loci and visualized the results using “RADStackshelpR” (DeRaad 2021). “vcfR” (Knaus and Grünwald 2017) was used to calculate summary statistics from output VCF files. First, *m* (the minimum coverage to create a stack) was iterated across 3 to 7, with default *M* and *n*; then, *M* (the maximum distance allowed between stacks within a locus) was iterated across 1 to 8, with optimal *m* (Figure S11) and default *n*; finally, *n* (the number of mismatches allowed between samples to build catalogs) was iterated across 2 to 4 with optimal *m* and *M*. Optimizations were conducted on 16 samples chosen at random and keeping all other parameters as the final Stacks run (*see below*). This process revealed that *m* = 3, *M* = 3, and *n* = 4 were optimal values for the structure of our data (Figure S11).

The RAD-seq reads were assembled using the de novo Stacks pipeline. Because the *in-silico* digestion showed the presence of paralogous loci, we implemented a strict haplotyping approach to prune those loci, following Piertney et al. (2023). The ustacks program was run with *m* = 3, *M* = 3, 2 maximum stacks per single de novo locus, 9 maximum mismatches allowed to align secondary reads to primary stacks, and a bounded model with an upper bound for the error rate (epsilon) of 0.05. The cstacks, sstacks, tsv2bam, and gstacks programs were run with default parameters, except that *n* = 4 was used in cstacks and gapped alignments between stacks were disabled in both cstacks and sstacks. After these steps, we removed from subsequent analyses 43 samples that showed low coverage (<10×; Figure S12a) and one outlier sample with a low number of genotypes (Figure S12b). We then integrated the de novo assembled loci with their alignment positions to the genome. Each locus was aligned to the reference genome (Marková et al. 2023) using BWA mem (Li, 2013) and the output was converted to BAM using samtools (Li et al. 2009). The stacks-integrate-alignments script was used to inject the alignment coordinates back into the Stacks output.

A final check was conducted at this stage to remove remaining paralogous loci. We first ran the populations program in Stacks to generate a filtered VCF file with the loci present in at least 50% of the individuals for each population, a minor allele frequency (MAF) of 0.01, and a maximum observed heterozygosity of 0.6. “HDPlot” (McKinney et al. 2017) was then run on the VCF file to identify the SNPs with an observed read ratio deviating from the expected for heterozygotes (1:1). We considered as putative paralogs all loci with at least one SNP with an absolute observed read ratio (|D|) above a stringent threshold of 6 (Figure S13). Additionally, we used VSEARCH to cluster loci by sequence similarity; all loci clustering with other loci were regarded as putative paralogs (as in Piertney et al. 2023). VSEARCH was run with an identity threshold of 0.1 (obtained via optimization across 0.1 to 0.9; the 0.1 identity threshold generated the least number of clusters, providing a conservative set of singleton loci; Piertney et al. 2023) with identity defined as the (matching columns)/(alignment length), no masking regions, rejection of the sequence match if the alignment begins with or contains gaps, and a complete database search. The paralogous loci identified by both methods (3,600; Figure S13) were blacklisted and removed from further analyses.

The population program was run again with the same options as before, except the paralogous loci were excluded from the report, and the MAF was 0.03. Finally, we conducted quality and completeness filtering of the SNP dataset using “SNPfiltR” (DeRaad 2022) and “vcfR”. First, we removed genotypes supported by a read depth below 5, quality below 30, and a heterozygote allele balance outside of the 0.1 to 0.9 range. We then applied a maximum depth cutoff to remove all SNPs with depth higher than double the mean depth of all SNPs (maximum depth cutoff = mean depth [60x] * 2 = 120x). Two samples had missing data >50% and were removed from further analyses. We then applied an 80% SNP completeness cutoff (ie 20% of missing data allowed per SNP) as a trade-off between minimizing the overall proportion of missing data across samples and maximizing the total number of loci (Figure S14). Duplicated SNPs (SNPs that were present twice in the VCF file) were also removed by retaining the one with the lowest amount of missing data. To reduce linkage disequilibrium between markers, the final VCF file was thinned to one SNP with the highest MAF for each RAD locus using custom scripts and VCFtools (Danecek et al. 2011). Finally, since “Scaffold_1610; HRSCAF = 1701” blasted to the mouse X chromosome, all SNPs in this scaffold (2,513) were removed to avoid ploidy differences between male and female individuals that may inflate subsequent population genomic analyses.

A total of 58,535 RAD SNP loci passed filtering and thinning, for a total of 154 remaining samples. The number of remaining samples for each of the 13 populations is presented in Table S5.

### Population structure

Genetic population structure was analyzed with the Admixture program (Alexander et al. 2009; Alexander and Lange 2011) using SNPs from RAD-seq. The filtered and thinned VCF file was converted to bed, and the additional files required, using PLINK (Chang et al. 2015). Admixture was run 10 times with random seeds and *K* = 1–13 to find the value with the lowest cross-validation error. For each value of K and each run, results were summarized and visualized using Pong (Behr et al. 2016).

### Genome-wide scan for selection and enrichment tests

We conducted genome-wide scans for adaptive genetic differentiation across populations using the Bayesian hierarchical method implemented in the BayPass program (Gautier 2015). BayPass was run with default settings on the RAD and CFH (CCP 20) SNP allele counts dataset. Genotype information was extracted for each RAD SNP and sample from the filtered and thinned VCF file using VCFtools and formatted for BayPass with the *gt.format* function in “rCNV” (Karunarathne et al. 2023). This was complemented with the CFH allele counts calculated for the biallelic SNPs identified in the CCP 20 domain (*see Results*). Six of the 20 SNPs in this domain were adjacent and occurred in the same variants and were therefore clumped together, for a total of 16 SNPs used in the analyses. To carry out our focal test on SNPs within CCP 20, we included all biallelic SNPs in this domain, rather than thinning them as we did for RAD markers. This approach is more robust compared to focusing on just the one SNP within CCP 20 that would remain after thinning; nonetheless, the one SNP within CCP 20 with the highest MAF (MAF = 0.5), which we would use in the analysis if we conducted thinning of CCP 20 SNPs with the same rationale as for RAD markers, was an outlier.

We first estimated the Ω matrix and the X^T^X statistic (an F_ST_-like statistic that accounts for shared population history; Günther and Coop 2013). Then, to identify X^T^X outliers, we simulated a pseudo-observed dataset (POD) containing 100,000 SNPs using parameters fixed to the posterior means of the original data. The POD was then analyzed with BayPass using the same settings as before to generate a null distribution of the X^T^X values. This distribution was then used to define 99% and 1% quantile thresholds. We considered as outliers under diversifying selection all SNPs with X^T^X values above the 99% quantile of the null (POD) distribution, and SNPs under balancing selection, those with X^T^X values below the 1% quantile.

To perform functional enrichment on the outlier SNPs (functional annotation clustering), we first used bedtools closest (Quinlan and Hall 2010) to search for the genes in the reference genome overlapping or nearest to each outlier SNP under diversifying or balancing selection; we then extracted the corresponding UniProt gene IDs from the annotations file and mapped them to the UniProtKB database on the UniProt website (https://www.uniprot.org/id-mapping). This list of gene IDs was used to conduct enrichment tests with David (Sherman et al. 2022). We used as background for the analysis the nearest or overlapping genes to all RAD SNPs (5,951 genes), which were extracted as explained above.

## Supplementary Material

msag082_Supplementary_Data

## Data Availability

Data and sequence alignments were made available on Figshare (https://doi.org/10.6084/m9.figshare.31266898). The RAD-seq data have been made available on NCBI Sequence Read Archive under BioProject PRJNA1366785 (SRA accession numbers: SRR36241184-SRR36241337).
