# Supplementary material for: A key regulator of missing-self innate immunity is polymorphic and under diversifying selection
Source: Mol Biol Evol. 2026 Apr 6;43(4):msag082. doi: 10.1093/molbev/msag082 (PMC13102179; doi:10.1093/molbev/msag082)
Supplement: msag082_Supplementary_Data [file msag082_supplementary_data.zip › Supplementary materials_revisions.docx]

**Supplementary materials for:**

**A key regulator of missing-self innate immunity is polymorphic and under diversifying selection**

*Rocco F. Notarnicola, Magdalena Herdegen-Radwan, Joanna Różańska-Wróbel, Mateusz Konczal, Karolina Przesmycka, Petr Kotlík, Wiesław Babik, Jacek Radwan*

**Table S1:** PAML model comparisons via likelihood ratio tests (LRT). ℓ_0_ = log-likelihood score of the null model; ℓ_1_ = log-likelihood score of the alternative model; df = degrees of freedom.

| Model comparison | Log-likelihood score (ℓ) | free parameters | df | LRT | P-value |
| --- | --- | --- | --- | --- | --- |
| **Partition 1 (1 - 633):** | | | | | |
| M0 vs M1a | ℓ_0_ = -13726.99 | 46 vs 47 | 1 | 800.99 | **3.279E-176** |
|  | ℓ_1_ = -13326.49 |  |  |  |  |
| M1a vs M2a | ℓ_0_ = -13326.49 | 47 vs 49 | 2 | 161.4 | **8.96E-36** |
|  | ℓ_1_ = -13245.79 |  |  |  |  |
| M7 vs M8 | ℓ_0_ = -13337.08 | 47 vs 49 | 2 | 181.29 | **4.29E-40** |
|  | ℓ_1_ = -13246.43 |  |  |  |  |
|  |  |  |  |  |  |
| **Partition 2 (633 - 1,243):** | | | | | |
| M0 vs M1a | ℓ_0_ = -13448.4 | 46 vs 47 | 1 | 582.04 | **1.35E-128** |
|  | ℓ_1_ = -13157.39 |  |  |  |  |
| M1a vs M2a | ℓ_0_ = -13157.39 | 47 vs 49 | 2 | 100.16 | **1.78E-22** |
|  | ℓ_1_ = -13107.3 |  |  |  |  |
| M7 vs M8 | ℓ_0_ = -13160.44 | 47 vs 49 | 2 | 115.77 | **7.24E-26** |
|  | ℓ_1_ = -13102.55 |  |  |  |  |

**Figure S2:** aBSREL results, partition 1. Node14 = the branch leading to bank voles; Node16 = the branch leading to v2 and v5 from v1; LRT = likelihood ratio test.

| Branch | Rates | Max. dN/dS | Test LRT | Uncorrected p-value |
| --- | --- | --- | --- | --- |
| N_galili | 2 | 3.07 (8.35%) | Not selected | for testing |
| **Node14** | **2** | **19.04 (7.92%)** | **41.21** | **<<0.0001** |
| M_musculus | 2 | 16.05 (4.25%) | Not selected | for testing |
| Node29 | 2 | 29.42 (3.22%) | Not selected | for testing |
| M_auratus | 2 | 123.95 (2.03%) | Not selected | for testing |
| P_roborovskii | 2 | 8.31 (4.15%) | Not selected | for testing |
| A_niloticus | 2 | 22.76 (3.51%) | Not selected | for testing |
| C_griseus | 1 | 0.48 (100.00%) | Not selected | for testing |
| A_sylvaticus | 2 | 31.67 (0.98%) | Not selected | for testing |
| Node38 | 2 | 3.40 (20.45%) | Not selected | for testing |
| P_maniculatus | 2 | 65.48 (3.38%) | Not selected | for testing |
| Node6 | 2 | 5.57 (18.93%) | Not selected | for testing |
| Node19 | 2 | 109.84 (1.05%) | Not selected | for testing |
| Node32 | 2 | 55.88 (1.73%) | Not selected | for testing |
| Node4 | 2 | 11.96 (10.86%) | Not selected | for testing |
| Node11 | 1 | 0.89 (100.00%) | Not selected | for testing |
| Node5 | 2 | 72.97 (4.01%) | Not selected | for testing |
| C_nivalis | 1 | 0.30 (100.00%) | Not selected | for testing |
| P_eremicus | 2 | 22.34 (1.95%) | Not selected | for testing |
| P_californicus | 1 | 0.59 (100.00%) | Not selected | for testing |
| A_amphibius | 2 | 24.73 (1.40%) | Not selected | for testing |
| Node1 | 1 | 0.35 (100.00%) | Not selected | for testing |
| Node24 | 2 | >1000 (0.28%) | Not selected | for testing |
| M_unguiculatus | 1 | 1.18 (100.00%) | Not selected | for testing |
| M_ochrogaster | 1 | 0.45 (100.00%) | Not selected | for testing |
| R_rattus | 1 | 0.46 (100.00%) | Not selected | for testing |
| Node28 | 1 | 0.36 (100.00%) | Not selected | for testing |
| P_obesus | 1 | 0.25 (100.00%) | Not selected | for testing |
| Node2 | 2 | 54.54 (2.68%) | Not selected | for testing |
| M_glareolus_V1 | 1 | 0.89 (100.00%) | 0 | 1 |
| M_oregoni | 1 | 0.54 (100.00%) | Not selected | for testing |
| Node21 | 1 | 0.73 (100.00%) | Not selected | for testing |
| M_pennsylvanicus | 1 | 0.43 (100.00%) | Not selected | for testing |
| **M_glareolus_V2** | **2** | **>1000 (0.19%)** | **6.78** | **0.01194** |
| M_glareolus_V5 | 1 | 1.85 (100.00%) | 0.77 | 0.28009 |
| Node34 | 1 | 0.46 (100.00%) | Not selected | for testing |
| Node3 | 1 | 2.10 (100.00%) | Not selected | for testing |
| R_norvegicus | 1 | 0.84 (100.00%) | Not selected | for testing |
| Node7 | 2 | 569.81 (1.05%) | Not selected | for testing |
| Node36 | 1 | >1000 (100.00%) | Not selected | for testing |
| Node16 | 1 | 0.80 (100.00%) | 0 | 1 |

**Figure S3:** aBSREL results, partition 2. Node5 = the branch leading to bank voles; Node6 = the branch leading to v1 and v5 from v2; LRT = likelihood ratio test.

| Branch | Rates | Max. dN/dS | Test LRT | Uncorrected p-value |
| --- | --- | --- | --- | --- |
| N_galili | 2 | >1000 (2.91%) | Not selected | for testing |
| Node29 | 2 | 19.84 (4.19%) | Not selected | for testing |
| M_auratus | 2 | 14.06 (2.56%) | Not selected | for testing |
| M_musculus | 2 | 10.33 (7.85%) | Not selected | for testing |
| **Node5** | **2** | **>1000 (2.20%)** | **11.53** | **0.00108** |
| A_sylvaticus | 2 | 1.55 (23.86%) | Not selected | for testing |
| Node10 | 2 | >1000 (2.34%) | Not selected | for testing |
| C_griseus | 2 | 97.16 (1.22%) | Not selected | for testing |
| P_roborovskii | 2 | 148.91 (0.27%) | Not selected | for testing |
| A_niloticus | 2 | 2.87 (24.92%) | Not selected | for testing |
| Node33 | 2 | 16.51 (2.25%) | Not selected | for testing |
| Node22 | 2 | 27.86 (2.60%) | Not selected | for testing |
| Node15 | 2 | 4.53 (20.27%) | Not selected | for testing |
| A_amphibius | 2 | >1000 (0.44%) | Not selected | for testing |
| Node32 | 2 | 4.77 (12.45%) | Not selected | for testing |
| R_norvegicus | 2 | 70.31 (1.59%) | Not selected | for testing |
| Node19 | 1 | 0.55 (100.00%) | Not selected | for testing |
| C_nivalis | 1 | 0.47 (100.00%) | Not selected | for testing |
| Node28 | 1 | 1.20 (100.00%) | Not selected | for testing |
| P_eremicus | 1 | 0.53 (100.00%) | Not selected | for testing |
| Node2 | 1 | 0.39 (100.00%) | Not selected | for testing |
| P_maniculatus | 1 | 0.44 (100.00%) | Not selected | for testing |
| R_rattus | 1 | 0.61 (100.00%) | Not selected | for testing |
| Node3 | 1 | 0.96 (100.00%) | Not selected | for testing |
| M_oregoni | 1 | 0.52 (100.00%) | Not selected | for testing |
| P_californicus | 1 | 1.15 (100.00%) | Not selected | for testing |
| M_unguiculatus | 1 | 0.29 (100.00%) | Not selected | for testing |
| Node4 | 2 | >1000 (0.80%) | Not selected | for testing |
| M_pennsylvanicus | 2 | 7.13 (5.67%) | Not selected | for testing |
| Node1 | 1 | 0.42 (100.00%) | Not selected | for testing |
| **M_glareolus_V1** | **2** | **43.76 (5.88%)** | **8.05** | **0.00627** |
| M_glareolus_V5 | 1 | 0.47 (100.00%) | 0 | 1 |
| M_ochrogaster | 2 | 135.99 (0.51%) | Not selected | for testing |
| P_obesus | 2 | 30.97 (1.51%) | Not selected | for testing |
| Node24 | 1 | 1.07 (100.00%) | Not selected | for testing |
| Node38 | 1 | 3.84 (100.00%) | Not selected | for testing |
| Node18 | 2 | 173.84 (1.91%) | Not selected | for testing |
| M_glareolus_V2 | 1 | 1.73 (100.00%) | 0.55 | 0.31868 |
| Node12 | 1 | 0.70 (100.00%) | Not selected | for testing |
| Node36 | 1 | >1000 (100.00%) | Not selected | for testing |
| Node6 | 1 | 1.34 (100.00%) | 0.09 | 0.4402 |

**Table S4:** Summary results from the population program in Stacks using all filtered, thinned SNPs. Pop ID: population ID; Num_Indv: average number of individuals at each SNP; P: mean frequency of the most common allele at each SNP; Obs_Het: mean observed heterozygosity; Obs_Hom: mean observed homozygosity; Exp_Het: mean expected heterozygosity; Exp_Hom: mean expected homozygosity; Pi: mean nucleotide diversity (π); Fis: mean inbreeding coefficient (F_IS_); StdErr: standard error; dab: Dąbrowica; TEL: Teleśnica; jul: Julianka; bial: Białystok; NIEP: Niepołomice; aug: Augustów; ol: Olsztyn; elk: Ełk; ziel: Zielonka; socz: Soczewka; zmig: Zmigród.

| Pop ID | Num_Indv | StdErr | P | StdErr | Obs_Het | StdErr | Obs_Hom | StdErr | Exp_Het | StdErr | Exp_Hom | StdErr | Pi | StdErr | Fis | StdErr |
| --- | --- | --- | --- | --- | --- | --- | --- | --- | --- | --- | --- | --- | --- | --- | --- | --- |
| dab | 17.62 | 0.00 | 0.83 | 0.00 | 0.24 | 0.00 | 0.76 | 0.00 | 0.24 | 0.00 | 0.76 | 0.00 | 0.24 | 0.00 | 0.02 | 0.00 |
| TEL | 12.28 | 0.00 | 0.83 | 0.00 | 0.24 | 0.00 | 0.76 | 0.00 | 0.24 | 0.00 | 0.76 | 0.00 | 0.25 | 0.00 | 0.03 | 0.00 |
| jul | 8.92 | 0.00 | 0.84 | 0.00 | 0.21 | 0.00 | 0.79 | 0.00 | 0.22 | 0.00 | 0.78 | 0.00 | 0.23 | 0.00 | 0.05 | 0.00 |
| bial | 10.31 | 0.00 | 0.84 | 0.00 | 0.24 | 0.00 | 0.76 | 0.00 | 0.23 | 0.00 | 0.77 | 0.00 | 0.24 | 0.00 | 0.01 | 0.00 |
| NIEP | 13.42 | 0.00 | 0.83 | 0.00 | 0.23 | 0.00 | 0.77 | 0.00 | 0.23 | 0.00 | 0.77 | 0.00 | 0.24 | 0.00 | 0.04 | 0.00 |
| brok | 10.81 | 0.00 | 0.84 | 0.00 | 0.24 | 0.00 | 0.76 | 0.00 | 0.23 | 0.00 | 0.77 | 0.00 | 0.24 | 0.00 | 0.02 | 0.00 |
| aug | 6.98 | 0.00 | 0.84 | 0.00 | 0.23 | 0.00 | 0.77 | 0.00 | 0.22 | 0.00 | 0.78 | 0.00 | 0.24 | 0.00 | 0.02 | 0.00 |
| ol | 10.99 | 0.00 | 0.84 | 0.00 | 0.23 | 0.00 | 0.77 | 0.00 | 0.23 | 0.00 | 0.77 | 0.00 | 0.24 | 0.00 | 0.03 | 0.00 |
| elk | 13.52 | 0.00 | 0.83 | 0.00 | 0.23 | 0.00 | 0.77 | 0.00 | 0.23 | 0.00 | 0.77 | 0.00 | 0.24 | 0.00 | 0.03 | 0.00 |
| ziel | 14.85 | 0.00 | 0.83 | 0.00 | 0.23 | 0.00 | 0.77 | 0.00 | 0.23 | 0.00 | 0.77 | 0.00 | 0.24 | 0.00 | 0.05 | 0.00 |
| gj | 5.69 | 0.00 | 0.84 | 0.00 | 0.22 | 0.00 | 0.78 | 0.00 | 0.21 | 0.00 | 0.79 | 0.00 | 0.23 | 0.00 | 0.03 | 0.00 |
| socz | 6.54 | 0.00 | 0.84 | 0.00 | 0.22 | 0.00 | 0.78 | 0.00 | 0.22 | 0.00 | 0.78 | 0.00 | 0.24 | 0.00 | 0.04 | 0.00 |
| zmig | 5.66 | 0.00 | 0.84 | 0.00 | 0.23 | 0.00 | 0.77 | 0.00 | 0.22 | 0.00 | 0.78 | 0.00 | 0.24 | 0.00 | 0.02 | 0.00 |

**Table S5:** Populations sampled for CFH and RAD sequencing. # samples = number of remaining samples per population after filtering of the RAD-seq data.

| Population | Longitude | Latitude | # samples |
| --- | --- | --- | --- |
| Augustów | N53.796200 | E23.150766 | 8 |
| Białystok | N53.279194 | E23.373034 | 11 |
| Brok | N52.700283 | E21.918842 | 12 |
| Dąbrowica | N50.475753 | E22.359139 | 22 |
| Ełk | N53.801549 | E22.399108 | 15 |
| Goly Jon | N53.693694 | E18.151994 | 6 |
| Julianka | N50.773350 | E19.465696 | 11 |
| Niepołomice | N50.00000 | E20.200000 | 14 |
| Olsztyn | N53.516669 | E20.624690 | 12 |
| Soczewka | N52.534904 | E19.598152 | 7 |
| Teleśnica | N49.220000 | E22.320000 | 14 |
| Zielonka | N52.582626 | E17.152514 | 16 |
| Zmigród | N51.508057 | E17.056494 | 6 |

**Table S6:** IDs and populations of origin of individuals sampled for full-length CFH sequencing.

| Bank vole ID | Population |
| --- | --- |
| R122 | Brok |
| R129 | Brok |
| R151 | Olsztyn |
| R152 | Olsztyn |
| R286 | Julianka |
| R304 | Żmigród |
| R306 | Żmigród |
| 009_22 | Ełk |
| 017_22 | Ełk |
| R149 | Olsztyn |
| R150 | Olsztyn |
| R262 | Goły Jon |
| R263 | Goły Jon |
| R264 | Goły Jon |
| R287 | Julianka |
| R288 | Julianka |
| R289 | Julianka |
| R303 | Żmigród |
| R305 | Żmigród |

**Table S7:** IDs and populations of the individuals sampled to generate a database of expressed CFH.

| Bank vole ID | Population ID | individuals/population |
| --- | --- | --- |
| R_4 | Brok | 9 |
| R_6 | Brok |  |
| R_2 | Brok |  |
| R_54 | Brok |  |
| R_55 | Brok |  |
| R_57 | Brok |  |
| R_58 | Brok |  |
| R_59 | Brok |  |
| R_60 | Brok |  |
| R_13 | Ełk | 12 |
| R_14 | Ełk |  |
| R_16 | Ełk |  |
| R_17 | Ełk |  |
| R_18 | Ełk |  |
| R_16 | Ełk |  |
| R_33 | Ełk |  |
| R_34 | Ełk |  |
| R_36 | Ełk |  |
| R_37 | Ełk |  |
| R_39 | Ełk |  |
| R_40 | Ełk |  |
| R_19 | Olsztyn | 11 |
| R_20 | Olsztyn |  |
| R_21 | Olsztyn |  |
| R_22 | Olsztyn |  |
| R_23 | Olsztyn |  |
| R_24 | Olsztyn |  |
| R_25 | Olsztyn |  |
| R_26 | Olsztyn |  |
| R_27 | Olsztyn |  |
| R_28 | Olsztyn |  |
| R_29 | Olsztyn |  |
| R_41 | Augustów | 6 |
| R_42 | Augustów |  |
| R_43 | Augustów |  |
| R_44 | Augustów |  |
| R_45 | Augustów |  |
| R_46 | Augustów |  |
| R_52 | Białystok | 2 |
| R_53 | Białystok |  |
| R_61 | Zielonka | 7 |
| R_62 | Zielonka |  |
| R_63 | Zielonka |  |
| R_66 | Zielonka |  |
| R_67 | Zielonka |  |
| R_68 | Zielonka |  |
| R_69 | Zielonka |  |
| R_8 | Żebra Żubra | 1 |
| R_9 | Dubeczno | 1 |
| R_11 | Dąbrowica | 1 |


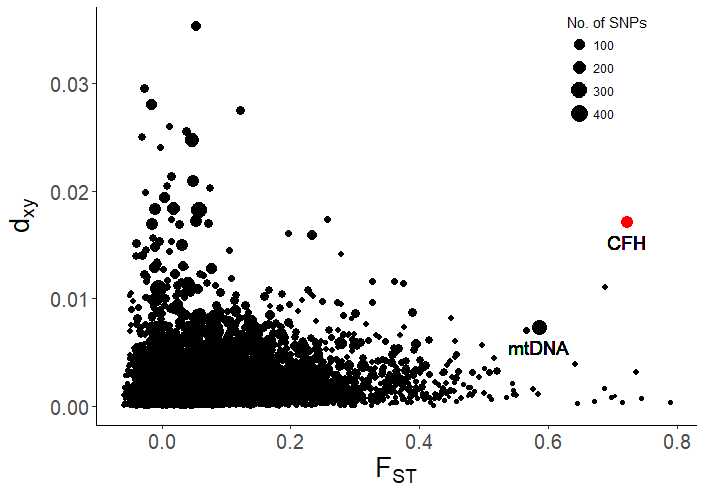


**Figure S1: CFH is among the most differentiated and diverged genes between Eastern and Western bank vole populations.**
Differentiation and divergence between Eastern (Białowieża) and Western (Włocławek) populations of bank voles. The plot is based on transcriptomic data from Niedziałkowska et al. (2023) which represent 10 individuals from Białowieża and 8 individuals from Włocławek populations. Each dot represents a single transcript. The transcript annotated as CFH is shown in red.


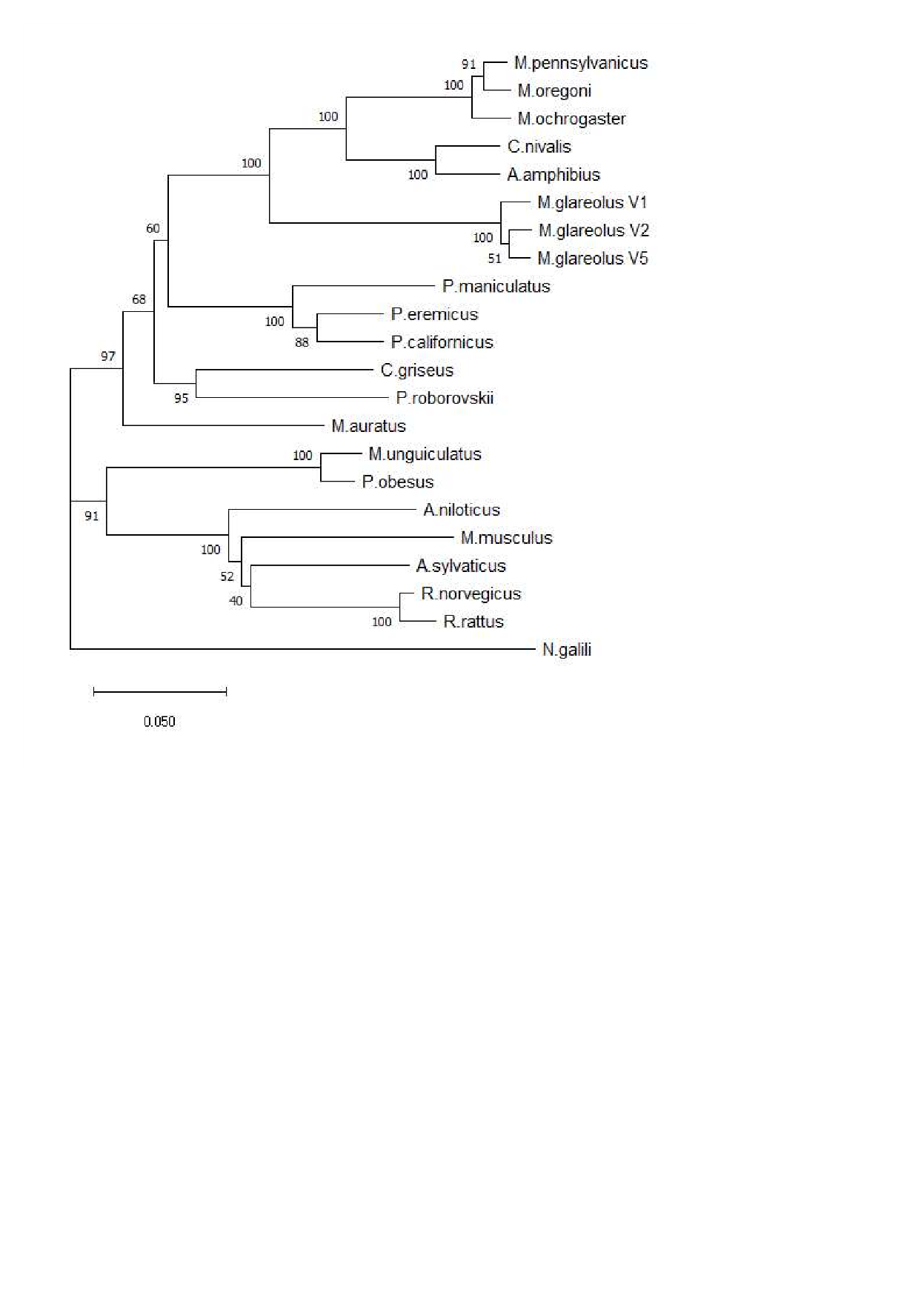


**A**

**Figure S2: CFH phylogenetic tree between *M. glareolus* variants, Cricetidae, *M. musculus*, and *R. norvegicus*.**CFH sequences from Cricetidae were obtained by blasting bank vole CFH v5 (‘jul_R289_v5’) to published Iso-seq databases (*M. pennsylvanicus*: SRR30640724; *P. maniculatus*: SRR29268902; *C. griseus*: DRR568658; *M. auratus*: SRR14718534). For *M. musculus* and *R. norvegicus* we used published CFH CDSs (BC066092-1, NM_130409-2, respectively). The tree was generated using MEGA11.


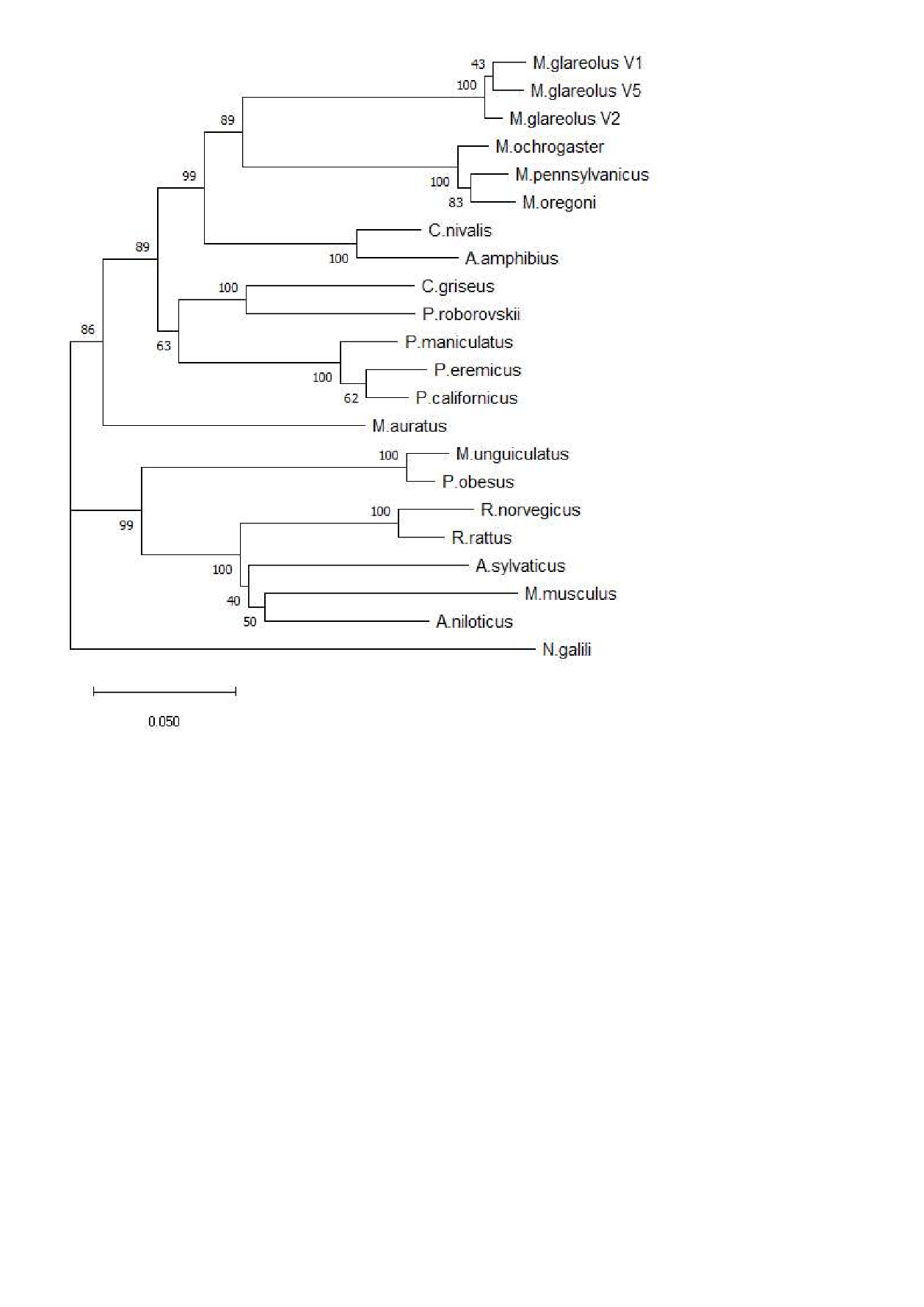


**B**

**Figure S2: Rodent phylogenetic tree from the two partitions of the multiple sequence alignment.**Maximum Likelihood (ML) tree of (a) partition 1 (site: 1 – 633) and (b) partition 2 (site: 634 – 1,243) of the multiple sequence alignment with rodent species. Numbers at nodes show bootstrap values. The sequences were aligned with Macse. The ML tree was generated with Mega 11 using a general time reversible model and a gamma distributed site-to-site rate variation. The two trees were independently used for the selection analyses with PAML, MEME, and aBSREL.


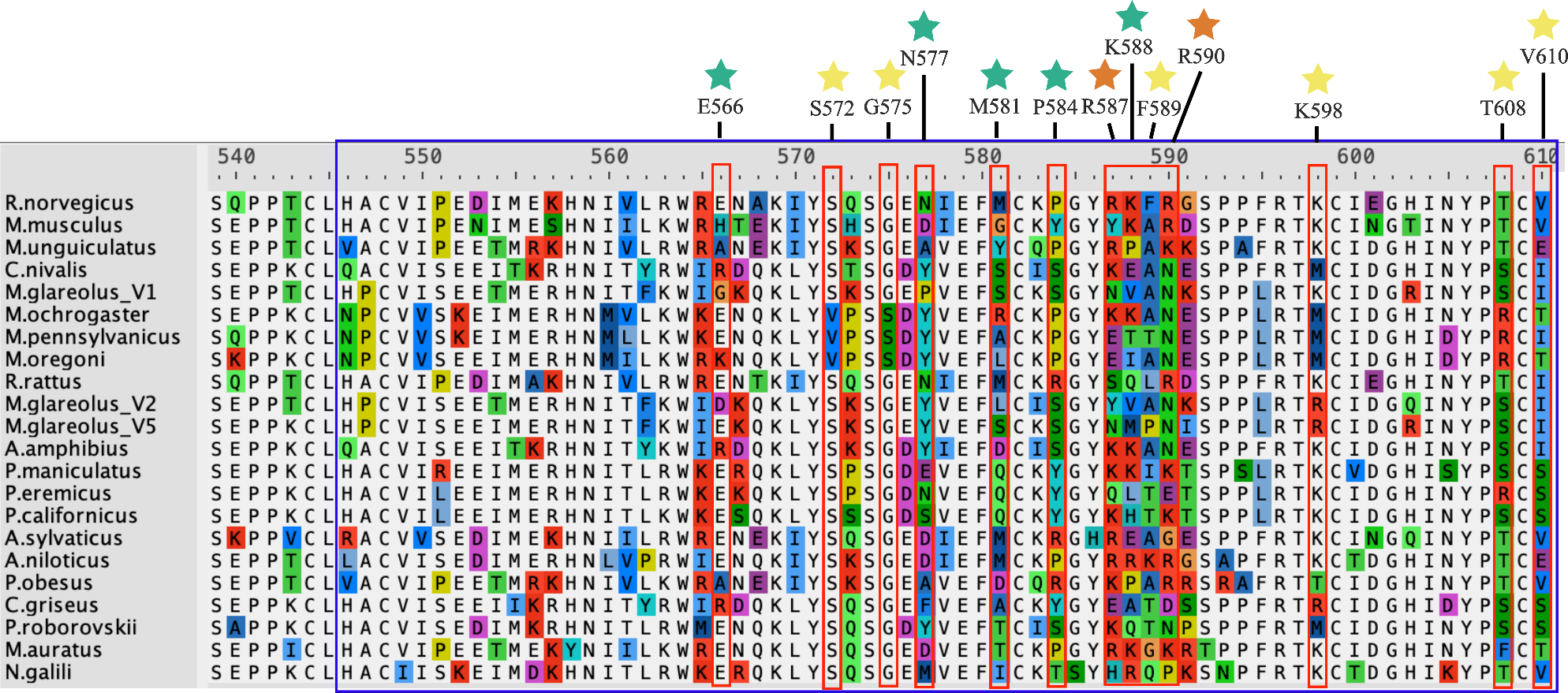


**Figure S3: Most of the sites under positive selection in partition 2 lie within CCP 20.**Amino acid multiple sequence alignment of the CCP 20 domain of CFH in rodents. The blue contour identifies the CCP 20 domain (546-610, partition 2). Differences from majority rule consensus of sites within each sequence are highlighted by colors. The red contours highlight sites with evidence of pervasive or episodic positive selection identified by PAML and/or MEME. These sites are also highlighted with stars: red-orange stars = sites identified by PAML; yellow stars = sites identified by MEME; green stars = sites identified by both PAML and MEME.


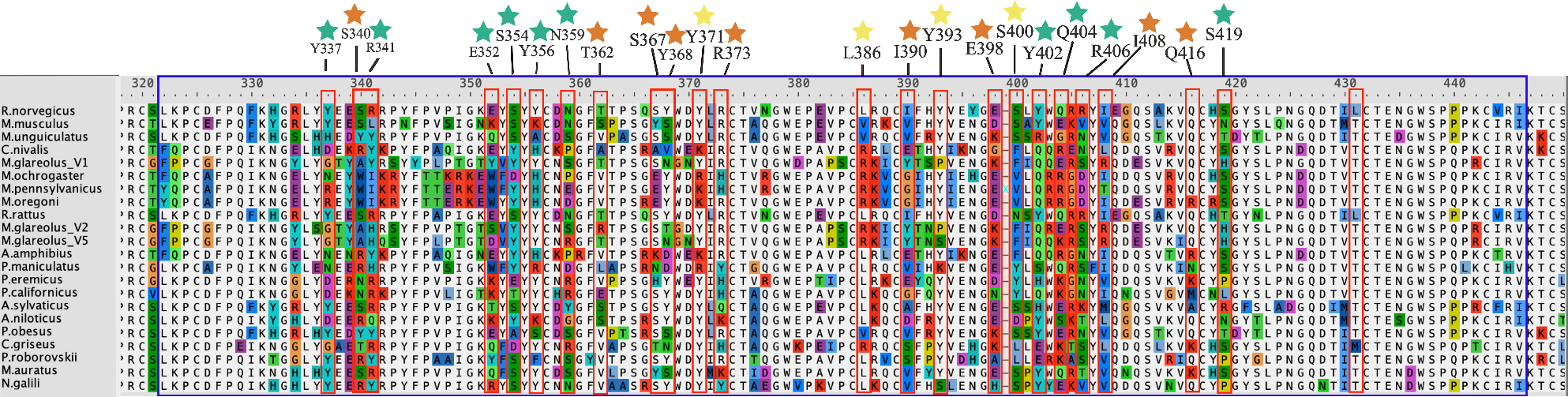


**Figure S4: Many sites in CCP 6 and 7 are under positive selection.**Amino acid multiple sequence alignment of the CCP 6 and 7 domains of CFH in rodents. The blue contour identifies the domains (322-446, partition 1). Differences from majority rule consensus of sites within each sequence are highlighted by colors. The red contours highlight sites with evidence of pervasive or episodic positive selection identified by PAML and/or MEME. These sites are also highlighted with stars: red-orange stars = sites identified by PAML; yellow stars = sites identified by MEME; green stars = sites identified by both PAML and MEME


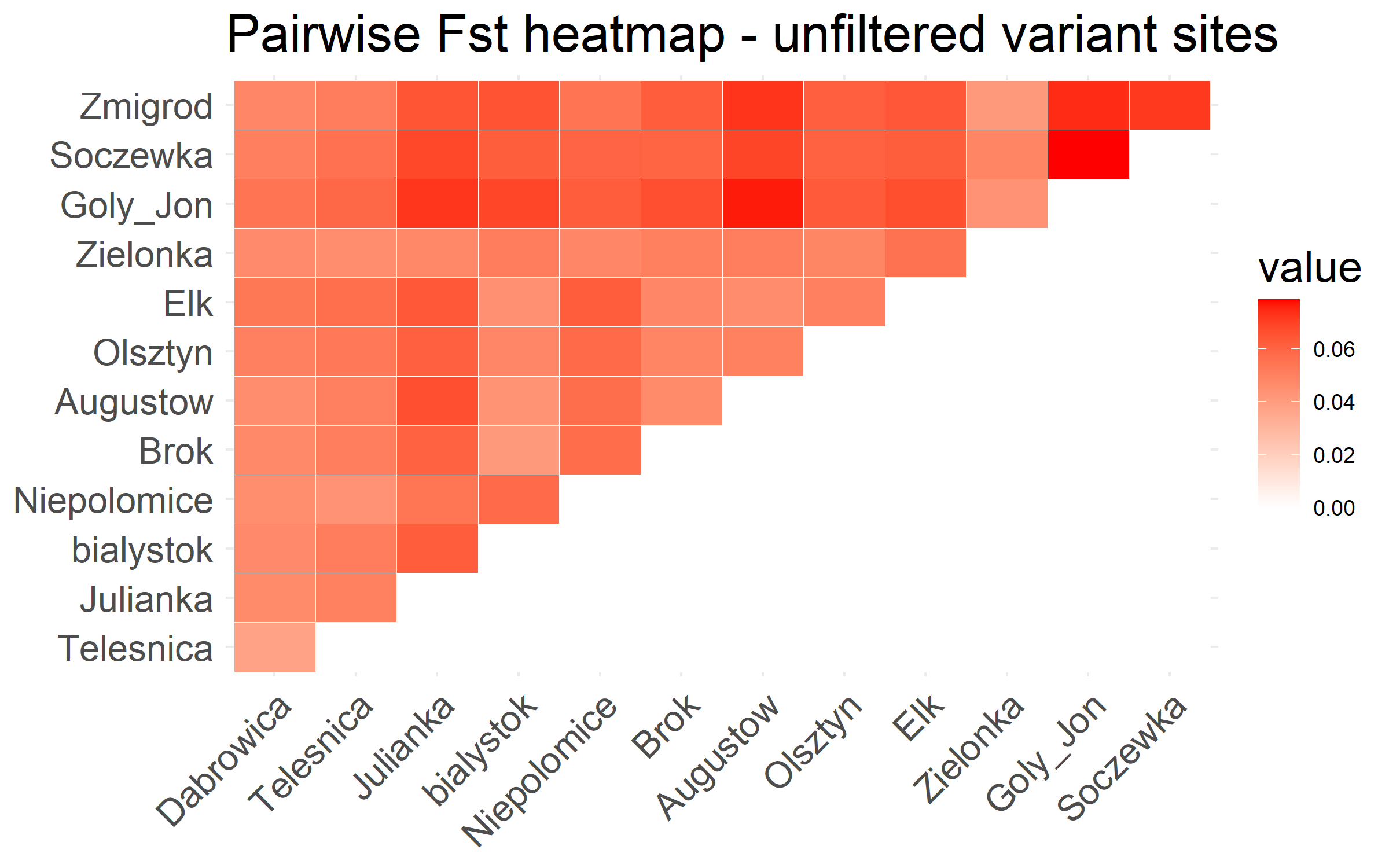


**Figure S5: Populations show reduced genomic differentiation.**Pairwise F_ST_ heatmap, generated using the results from the population program in Stacks using all filtered, thinned SNPs.

*
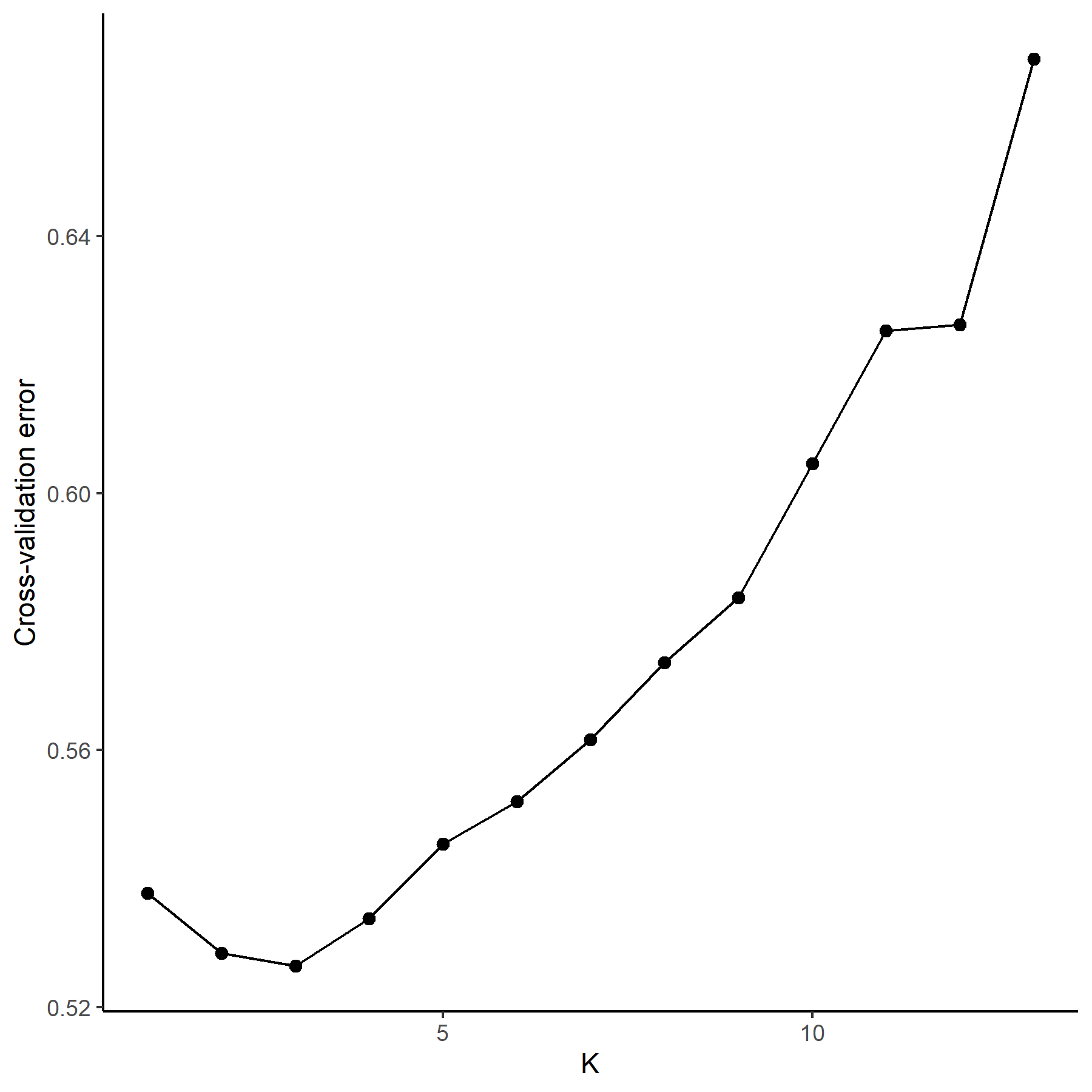
*

**Figure S6: Populations cluster in three main groups.**X-axis: number of groups (K); y-axis: cross-validation error.


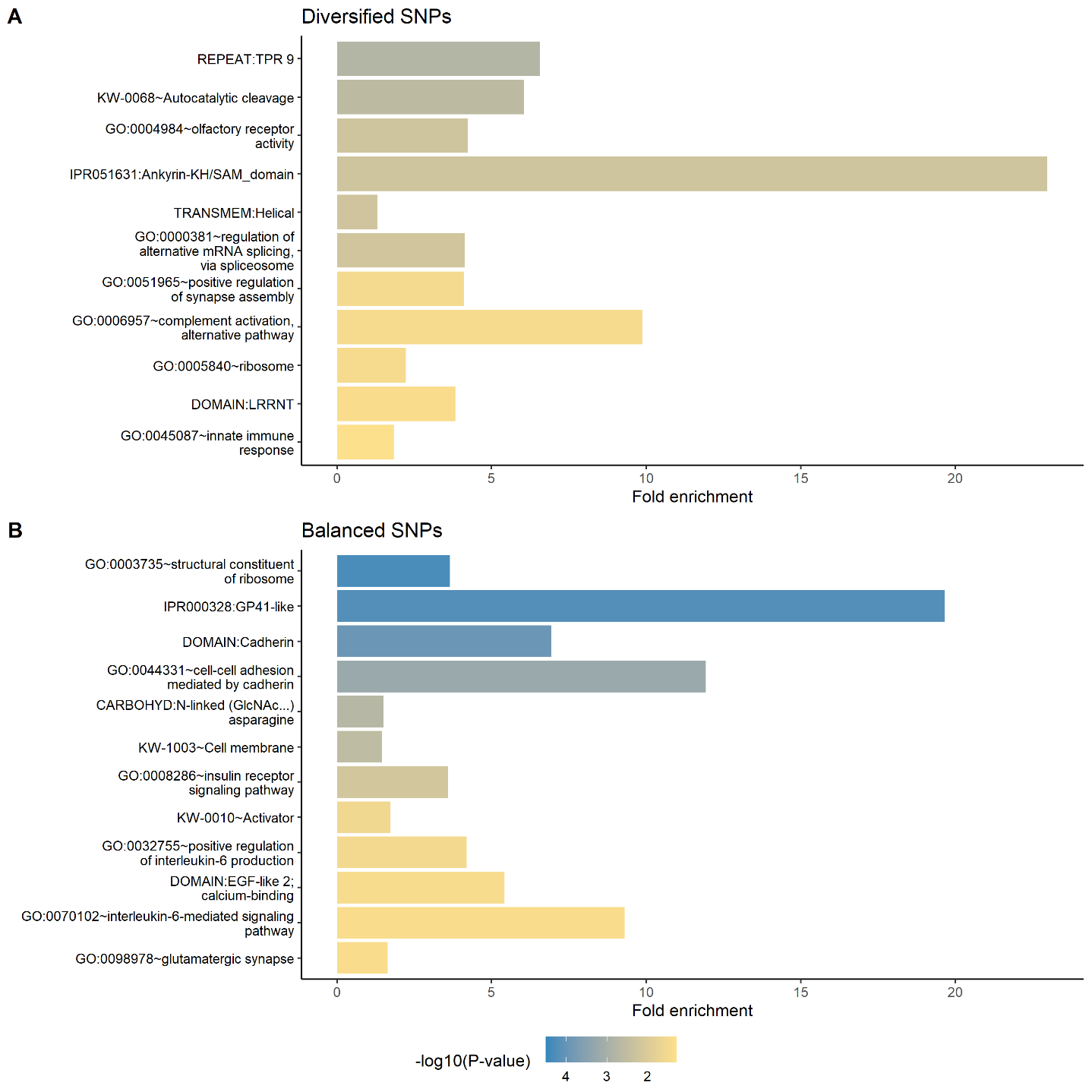


**Figure S7: SNPs under positive selection are enriched for terms related to alternative complement activation and innate immunity, while those under balancing selection for terms related to Interleukin 6 pathways.**Enrichment tests using the overlapping or closest genes to the SNPs under diversifying (a) or balancing (b) selection. X-axis: fold enrichment; colors of the bars: -log_10_ of p-values. Enriched terms are clustered based on function; one representative term for each cluster is shown.


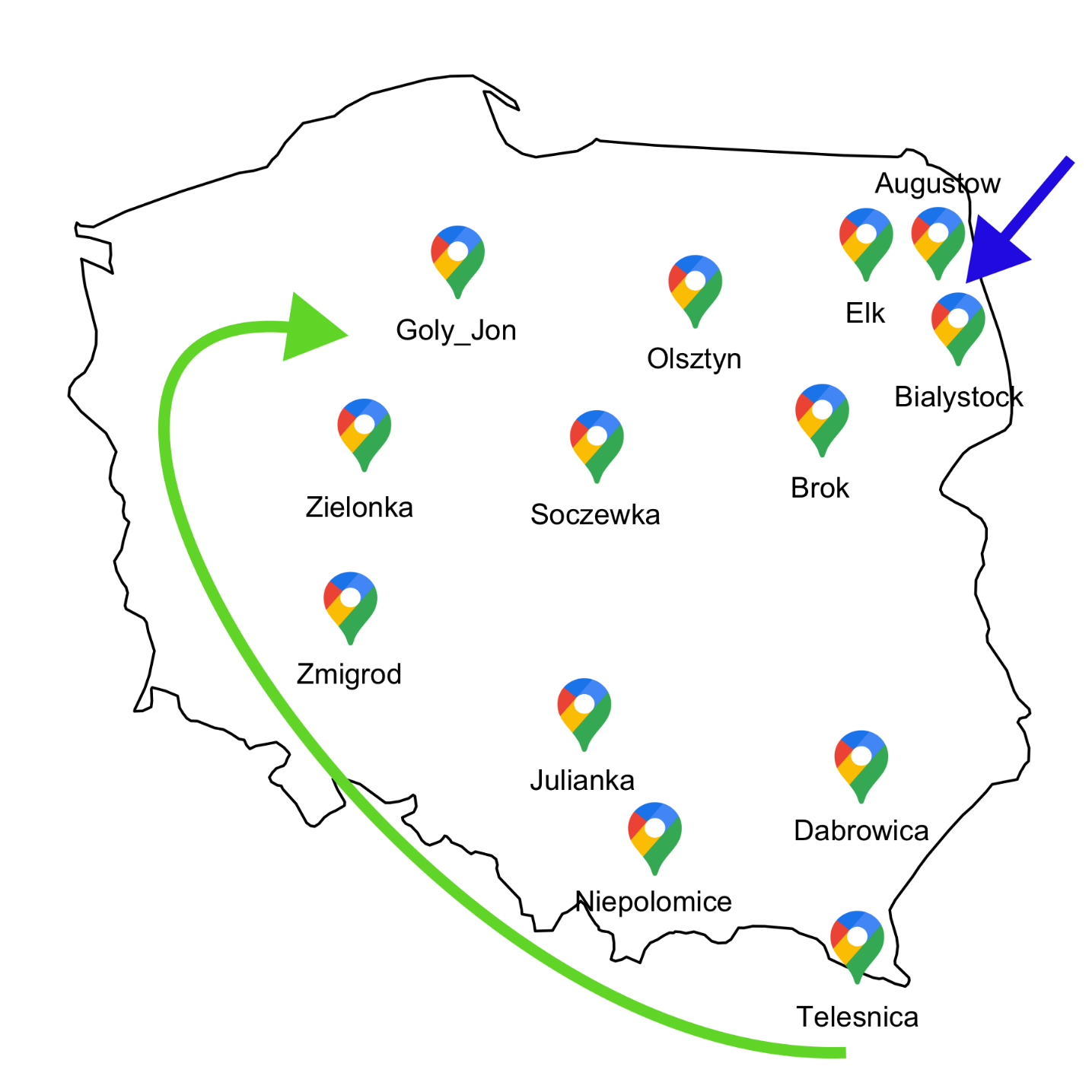


**Figure S8: The sampled populations span the postglacial colonization areas and the contact zone of the Carpathian and the Eastern bank vole lineages.**Map of Poland showing the location of the populations sampled for CFH and RAD sequencing. Green arrow: postglacial colonization area of the Carpathian clade; blue arrow: postglacial colonization area of the Eastern clade.


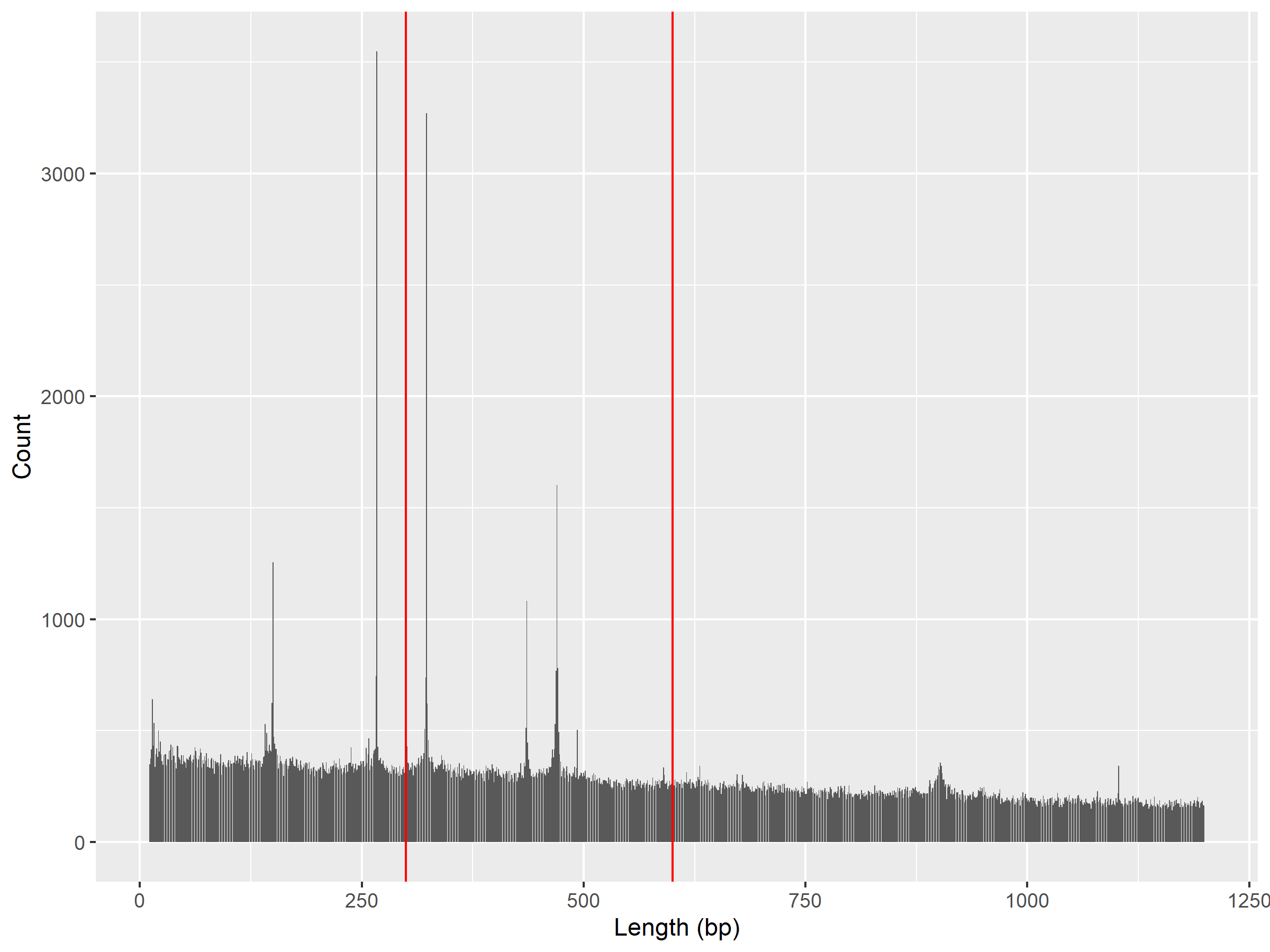


**Figure S9: *In-silico* digestion of the genome.**X-axis: lengths (bp) of the fragments cut with the 3 restriction enzymes *XbaI*, *EcoRI*, and *NheI*. Red lines show the selected size range (300-600 bp) for RAD-seq. The genome used has accession: GCF_902806735.1.


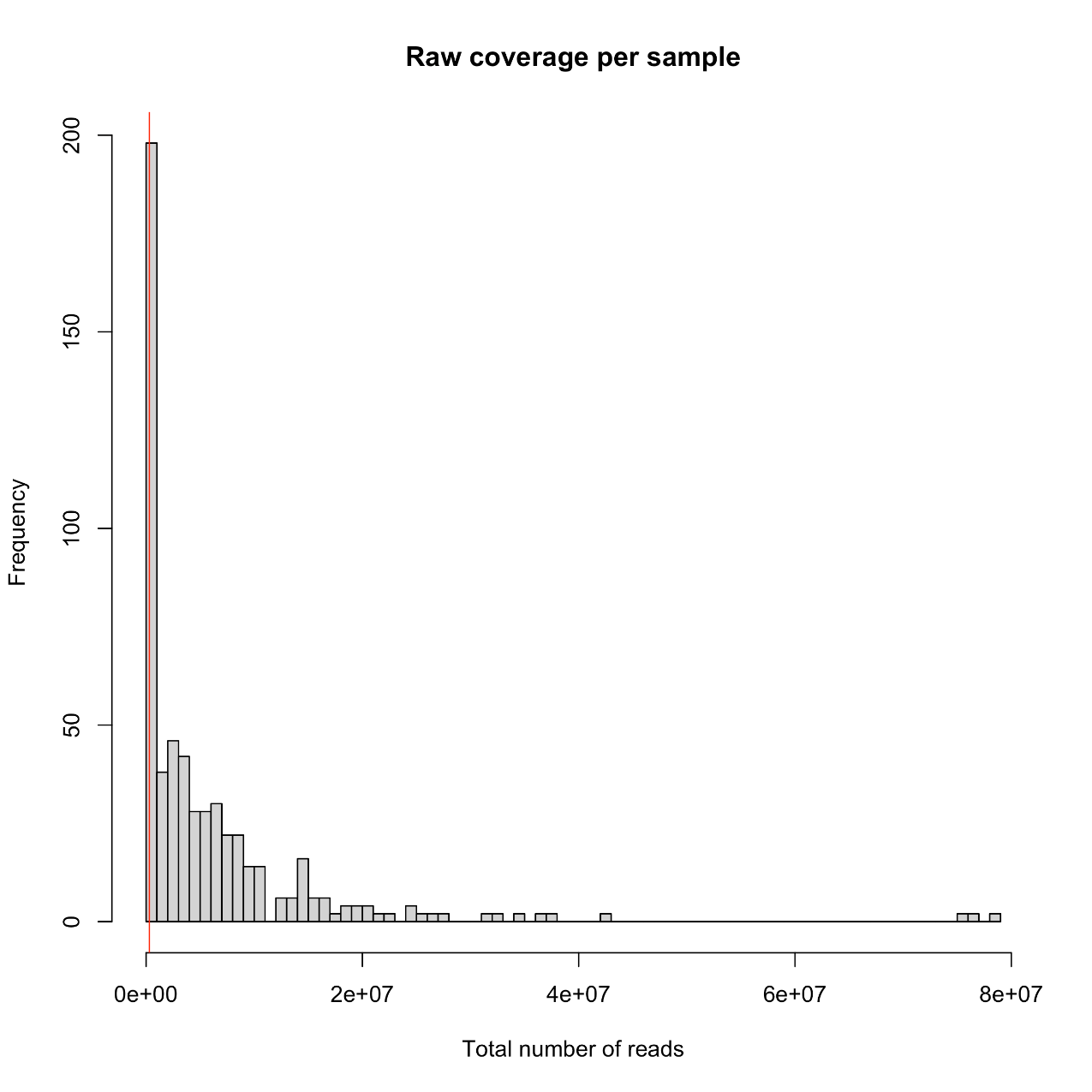


**Figure S10: Histogram of the raw coverages per sample.**
The red bar shows 10% of the median of the raw coverage across all samples (= 302,396.5 reads). The samples below this threshold were discarded. Coverages were calculated for each forward and reverse file of each sample.


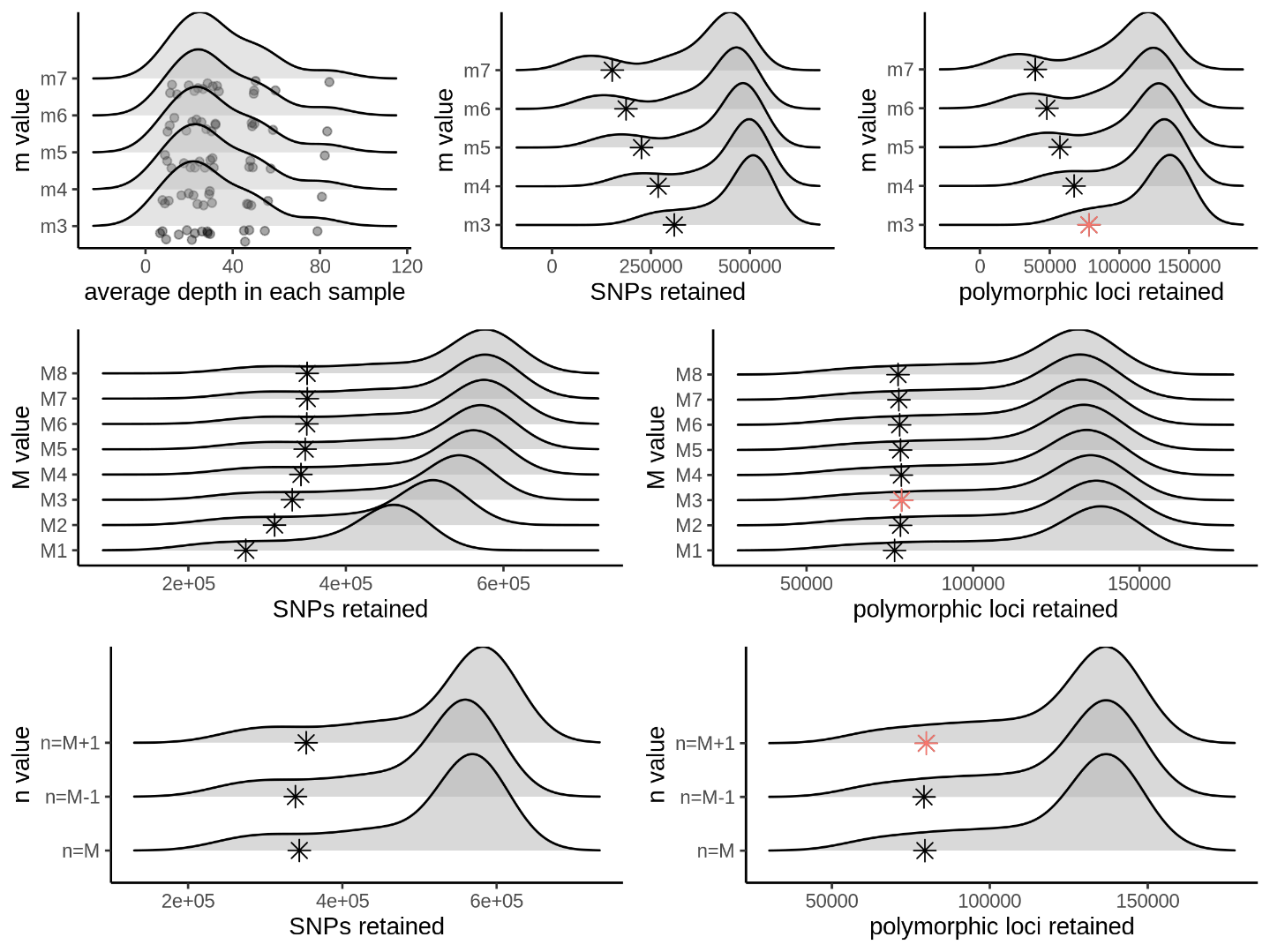


**Figure S11: Stacks parameter optimizations.**(Top) *m* parameter optimization shown in density plots of the average depth in each sample (left), number of Single Nucleotide Polymorphisms (SNPs) retained (middle), and number of polymorphic sites retained (right) at each value of *m* = 3-7. (Middle) *M* parameter optimization shown in density plots of the number of SNPs retained (left) and the number of polymorphic loci retained (right) for each value of *M* = 1-8. (Bottom) *n* parameter optimization shown in density plots of the number of SNPs retained (left) and the number of polymorphic loci retained (right) for each value of *n*: *n* = *M* = 3; *n* = *M* – 1 = 2; *n* = *M* + 1 = 4. The red stars in the rightmost plots show the optimal value for each parameter.


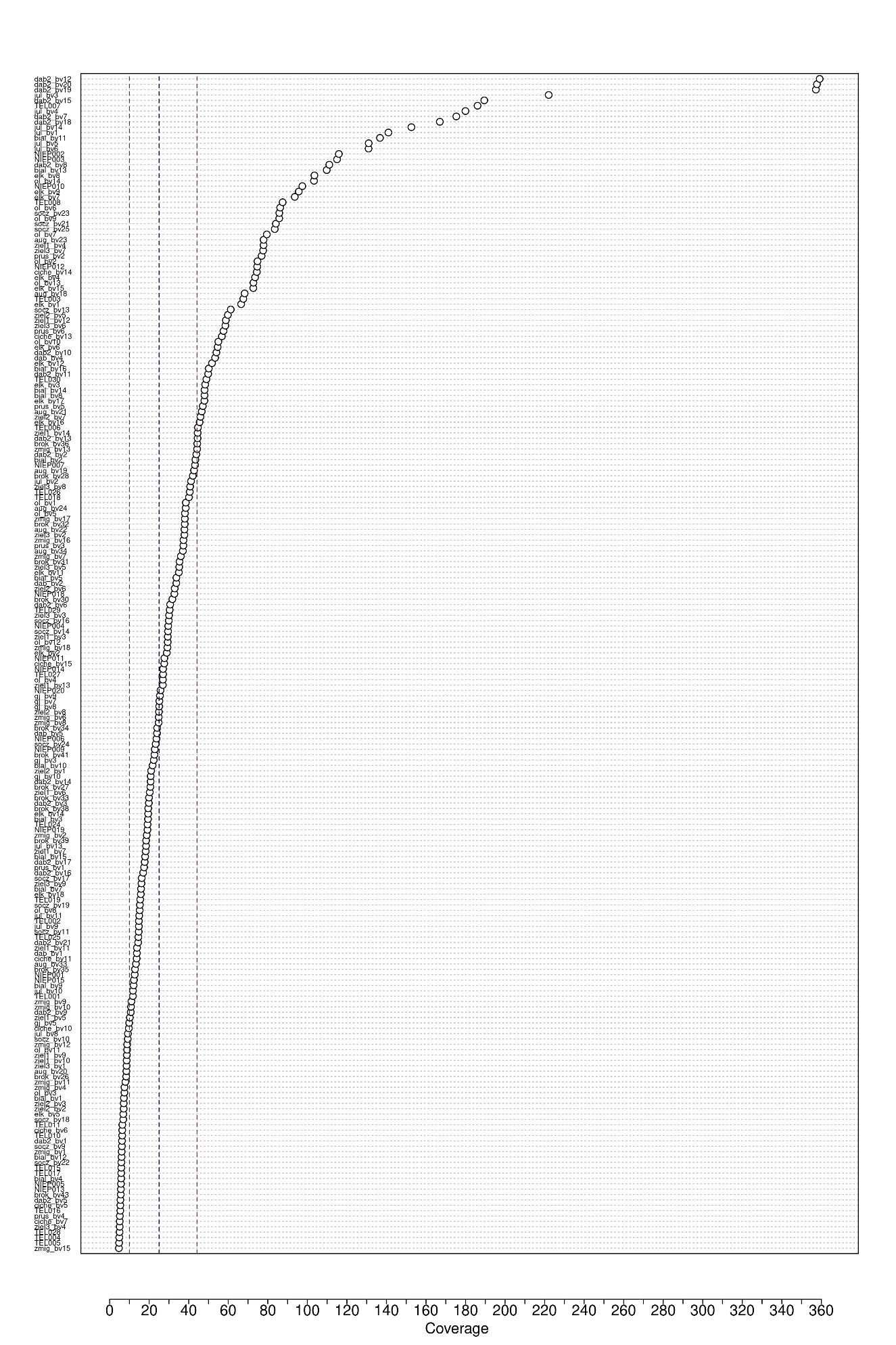


**A**


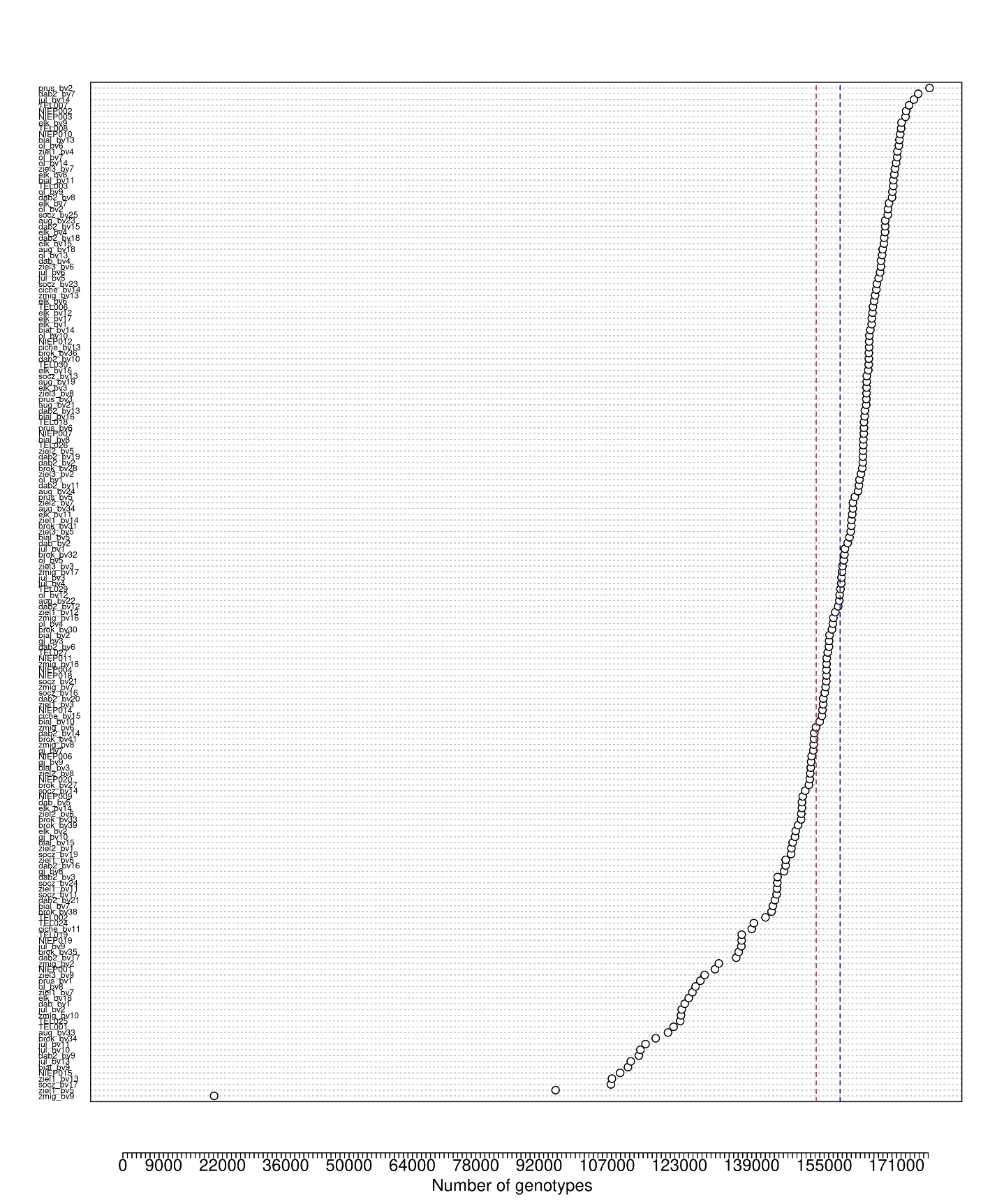


**Figure S12: Sample coverages and number of genotypes after the de novo Stacks pipeline.**(A) Coverages per sample. X-axis = coverage adjusted by number of samples present at a locus. Green line = 10x coverage used as cutoff (43 samples below this were discarded); blue line = 25x coverage; red line = mean coverage. Samples ordered by coverage. (B) Number of genotypes for each sample. Red line = mean number of genotypes; blue line = median number of genotypes. Only the samples that passed the coverage cut-off are shown. The sample at the bottom (‘zmig_bv9’) was an outlier with too few genotypes and was discarded. Samples ordered by number of genotypes.

**B**

**Figure S10: Sample coverages and number of genotypes after the *de novo* Stacks pipeline.**(A) Coverages per sample. X-axis = coverage adjusted by number of samples present at a locus. Green line = 10x coverage used as cutoff (43 samples below this were discarded); blue line = 25x coverage; red line = mean coverage. Samples ordered by coverage. (B) Number of genotypes for each sample. Red line = mean number of genotypes; blue line = median number of genotypes. Only the samples that passed the coverage cut-off are shown. The sample at the bottom (‘zmig_bv9’) was an outlier with too few genotypes and was discarded. Samples ordered by number of genotypes.


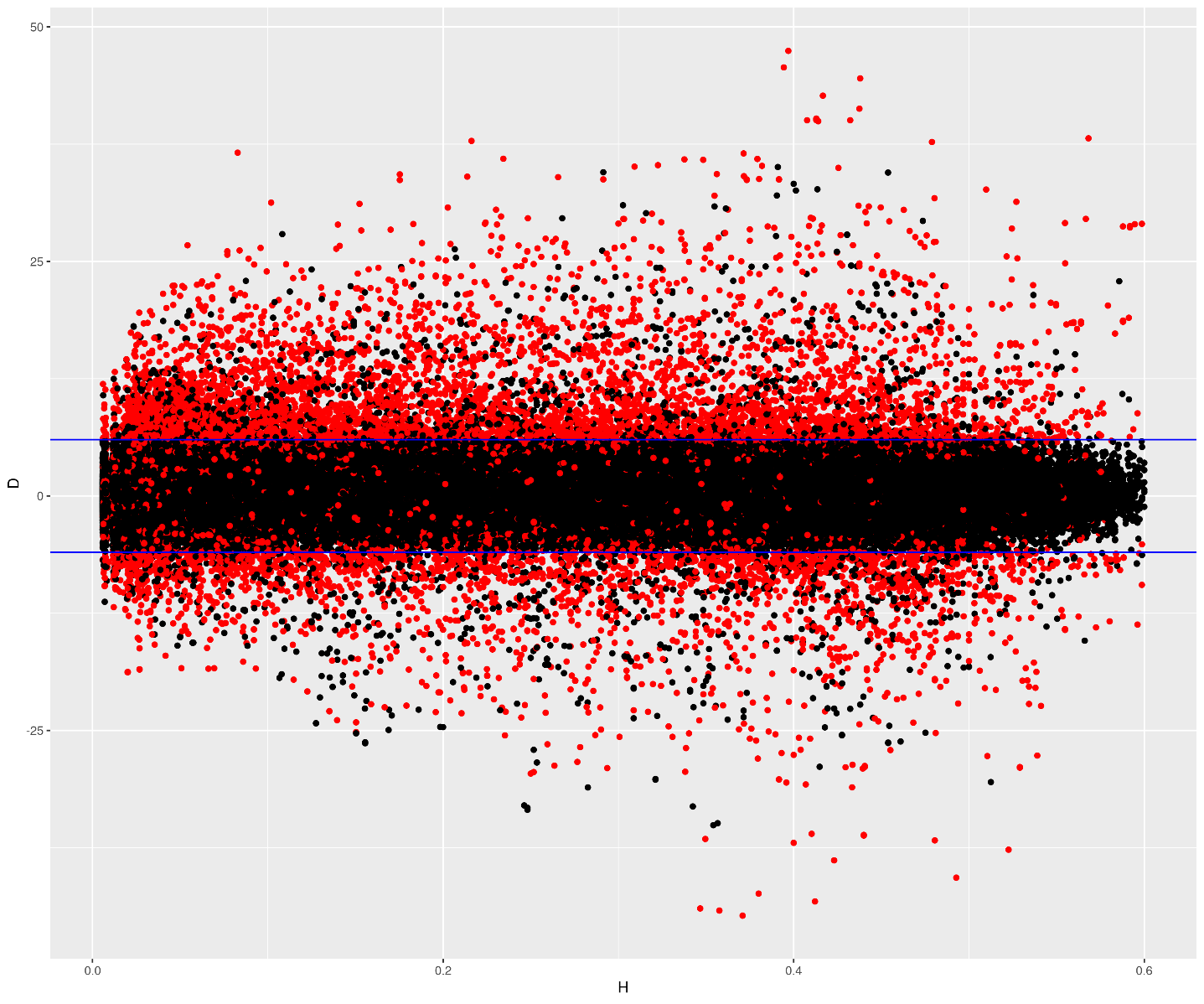


**Figure S13: Paralogous loci check.**The graph shows the results of ‘HDPLOT’. X-axis = expected proportion of heterozygous individuals; y-axis = deviation from the 1:1 allelic ratio for heterozygotes. Blue lines = chosen cut-off for the read ratio deviation (|D| = 6). Black dots = SNPs in non-paralogous loci; red dots = SNPs in paralogous loci identified by both ‘HDPlot’ and VSEARCH (3,600 loci, discarded from further analyses). Loci with at least one SNP with |D| >6 in ‘HDPlot’ were considered paralogous; that is why some SNPs were discarded even though they had |D| <6.


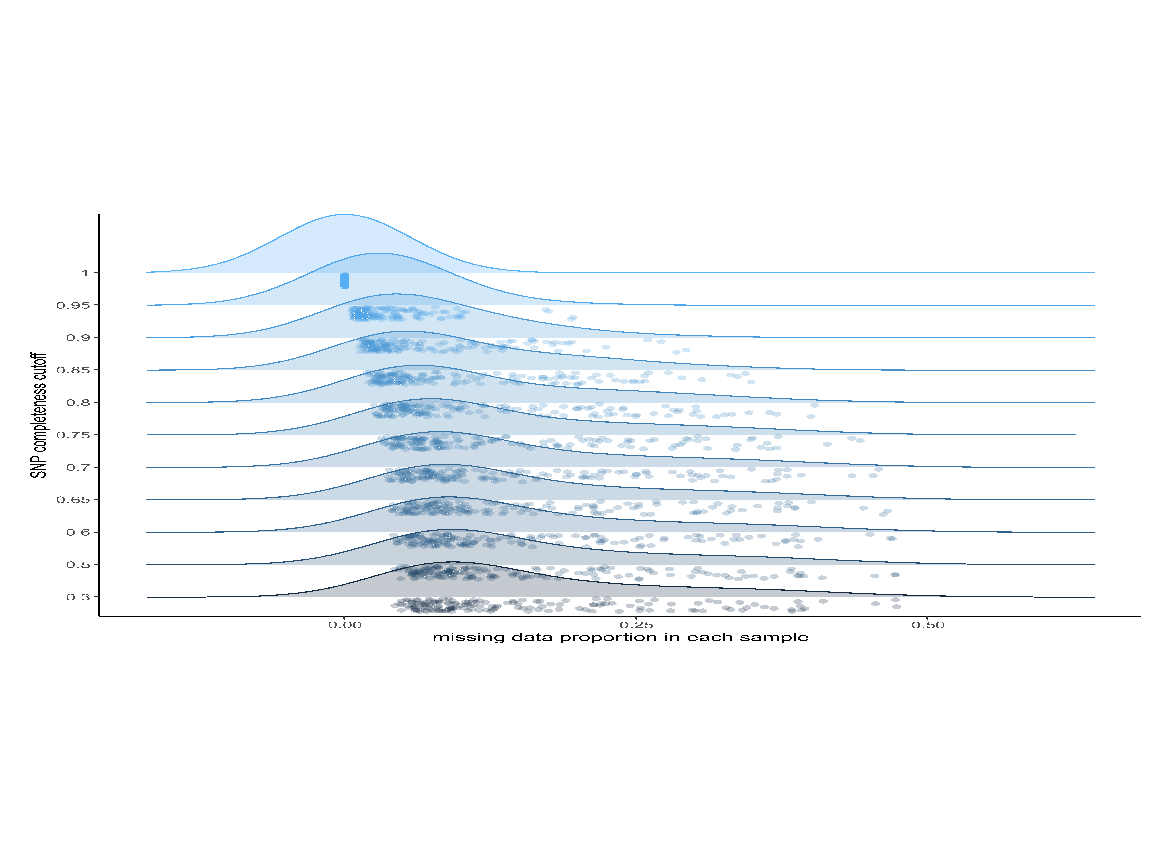

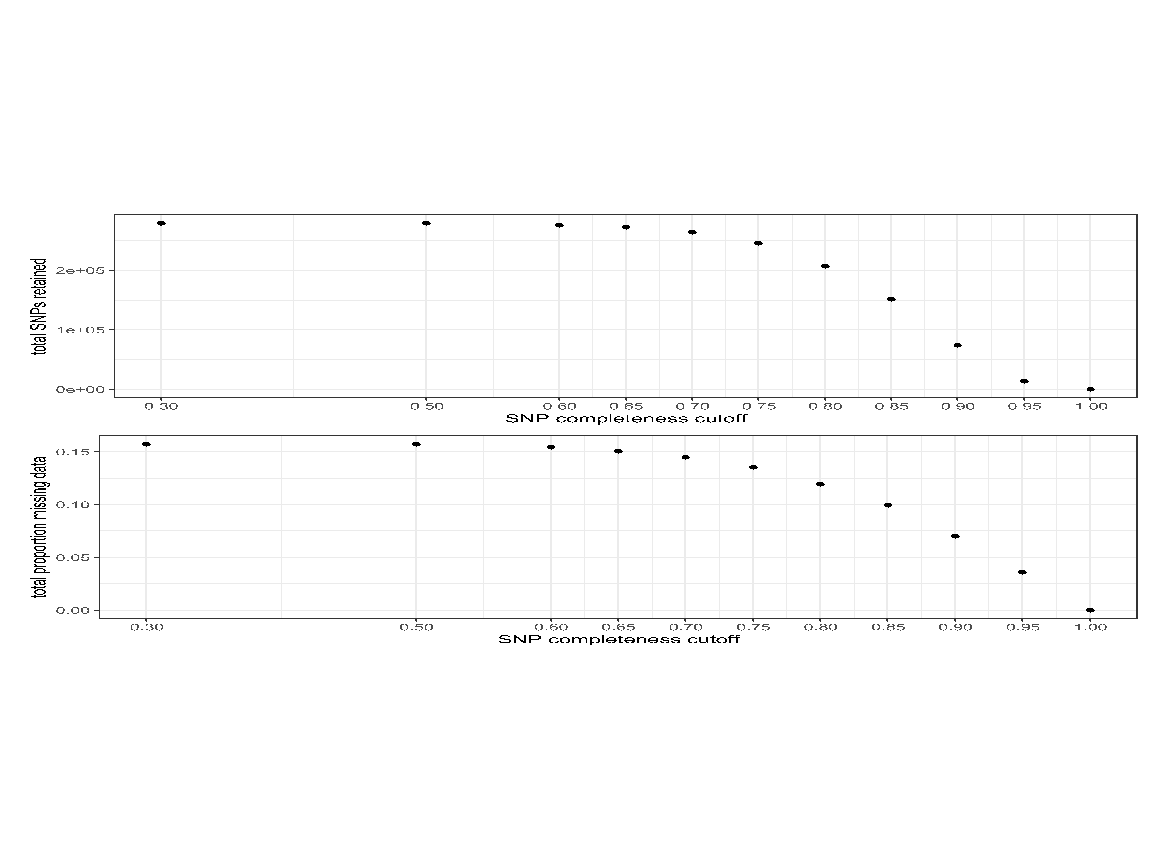


**A**

**B**

**Figure S14: SNP completeness cutoff effects on missing data and number of SNPs.**(A) Density plot showing the proportion of missing data per sample for each SNP completeness (the opposite of missing data per SNP) cutoff (0.3-1). Dots are samples. (B) Total number of SNPs retained and total proportion of missing data by SNP completeness cutoff. The chosen cutoff (0.8) represents a tradeoff between reducing the differences in missing data between samples and retaining the most SNPs.
